# Macroscale Gradient‐Informed Neural Oscillation Topography in Parkinson's Disease

**DOI:** 10.1002/mds.70277

**Published:** 2026-03-16

**Authors:** Hao Ding, Ke Xie, Manuel Bange, Hannah Kühne, Jenny Blech, Bahman Nasseroleslami, Jens Volkmann, Sergiu Groppa, Muthuraman Muthuraman

**Affiliations:** ^1^ Department of Neurology University Hospital Würzburg Würzburg Germany; ^2^ Academic Unit of Neurology Trinity College Dublin Dublin Ireland; ^3^ McConnell Brain Imaging Centre, Montreal Neurological Institute and Hospital McGill University Montreal Quebec Canada; ^4^ Institute of Computer Science University Augsburg Augsburg Germany; ^5^ Department of Neurology University Medical Center Mainz Mainz Germany; ^6^ Department of Neurology Saarland University Hospital Homburg Germany

**Keywords:** Parkinson's Disease, functional gradients, neural oscillatory topography

## Abstract

**Background:**

Parkinson's disease (PD) is characterized by large‐scale disruptions in beta and gamma oscillations. Although subcortical beta power is an established biomarker for current adaptive deep brain stimulation (aDBS), it may not fully capture the global pathophysiological burden and the macroscale hierarchical reorganization of the cortex.

**Objective:**

We characterize the frequency‐specific reorganization of the cortical hierarchy across resting and motor states using functional gradients. We sought to identify topographic biomarkers that emerge across different behavioral states and determine whether these hierarchical features provide predictive power for global motor severity.

**Methods:**

High‐density electroencephalography and magnetic resonance imaging–based source reconstruction were employed in patients with PD (n = 35) and healthy control subjects (n = 34). To characterize cortical connectivity transitions, we applied a manifold learning framework to derive frequency‐specific functional gradients. We quantified the diagnostic and predictive utility of these hierarchical features and performed transcriptomic enrichment analysis to validate the biological relevance of the alterations.

**Results:**

Patients with PD exhibited a macroscale reorganization of the cortical hierarchy that was both frequency specific and state dependent. These gradient‐based biomarkers effectively differentiated patient groups and significantly predicted global Unified Parkinson's Disease Rating Scale Part III severity. Findings showed a robust framework with distinct topographical signatures, manifesting as a redistribution of informative signals across cortical regions.

**Conclusions:**

This work demonstrates that PD induces a macroscale reorganization of the cortical hierarchy. State‐dependent topographical biomarkers effectively predict clinical severity and align with the disease pathological landscape. By identifying optimal sensing sites across distributed networks, our findings provide a principled reference to support next‐generation, cortical‐guided aDBS. © 2026 The Author(s). *Movement Disorders* published by Wiley Periodicals LLC on behalf of International Parkinson and Movement Disorder Society.

Parkinson's disease (PD) is characterized by progressive dopaminergic loss and altered neural oscillations.^1^ Although hallmark motor symptoms (bradykinesia, rigidity, tremor) are central to the diagnosis, a wide range of nonmotor symptoms also contribute significantly to the clinical syndrome, reflecting more widespread neurodegeneration.

A key pathophysiological feature of PD is altered neural oscillatory activity. Specifically, changes in the beta and gamma bands are closely linked to motor dysfunctions, while alpha‐band alterations are increasingly recognized for their association with nonmotor and cognitive symptoms.^2^, ^3^, ^4^ Recent advancements in deep brain stimulation (DBS) have leveraged these oscillatory features, specifically in subcortical local field potentials (LFPs), to develop adaptive DBS (aDBS).^5^ However, the dynamic and state‐dependent nature of PD symptoms, coupled with significant individual variability, suggests that subcortical LFP signals alone may not provide a comprehensive biomarker for effective aDBS. This is mainly because subcortical signals have restricted spatial information and may not be able to capture the full spectrum of the disease symptoms.^6^ This has led to growing interest in expanding biomarker discovery to include broader cortical networks and their dynamic interactions.

Cortical dynamics, particularly when analyzed in relation to their spatial organization, offer valuable insights for identifying biomarkers in PD. For example, evidence shows that cortical beta hypersynchrony is attenuated by STN‐DBS,^2^ and cortical gamma oscillations may serve as markers for dyskinesia and gait.^3^, ^7^, ^8^ Although these findings emphasize the primary motor cortex as a key region of interest, they open a vital avenue for expanding the exploratory scope beyond a single region to understand broader cortical networks. The human cortex exhibits a hierarchical organization, with regions varying in structure and connectivity in a gradual, orderly manner.^9^, ^10^ These variations form well‐defined cortical gradients that reflect a fundamental principle of brain architecture: a continuous functional transition from unimodal sensory and motor regions to transmodal regions supporting higher‐order cognitive function. These gradients are consistent with key neuroanatomical principles, including myelination, cortical thickness, and laminar differentiation, and are robust across various brain states.^11^, ^12^, ^13^, ^14^ In PD, alterations have been observed within these cortical hierarchies, and these changes are strongly associated with motor symptoms.^15^, ^16^ This led us to hypothesize that cortical gradients offer a robust biomarker for PD, because they maintain topological stability across brain states while remaining sensitive enough to capture task‐specific dynamics, providing a more reliable input for aDBS.

To investigate these disruptions, we leveraged electroencephalography (EEG) to probe cortical dynamics across multiple frequency bands. We employed an established dimensionality reduction framework to identify principal cortical gradients,^17^ which represent the topographical organization and smooth transitions of connectivity profiles across the cortex. We compared these gradient features against traditional regional band‐power biomarkers and validated their clinical utility by predicting motor symptom severity. This disease relevance was further confirmed through an enrichment analysis with associated gene clusters. Ultimately, we argue that this work shows a hierarchical, stable, and task‐sensitive alteration in cortical organization, providing a novel framework to inform the future development of cortical‐guided aDBS (Fig. 1).

**FIG. 1:**
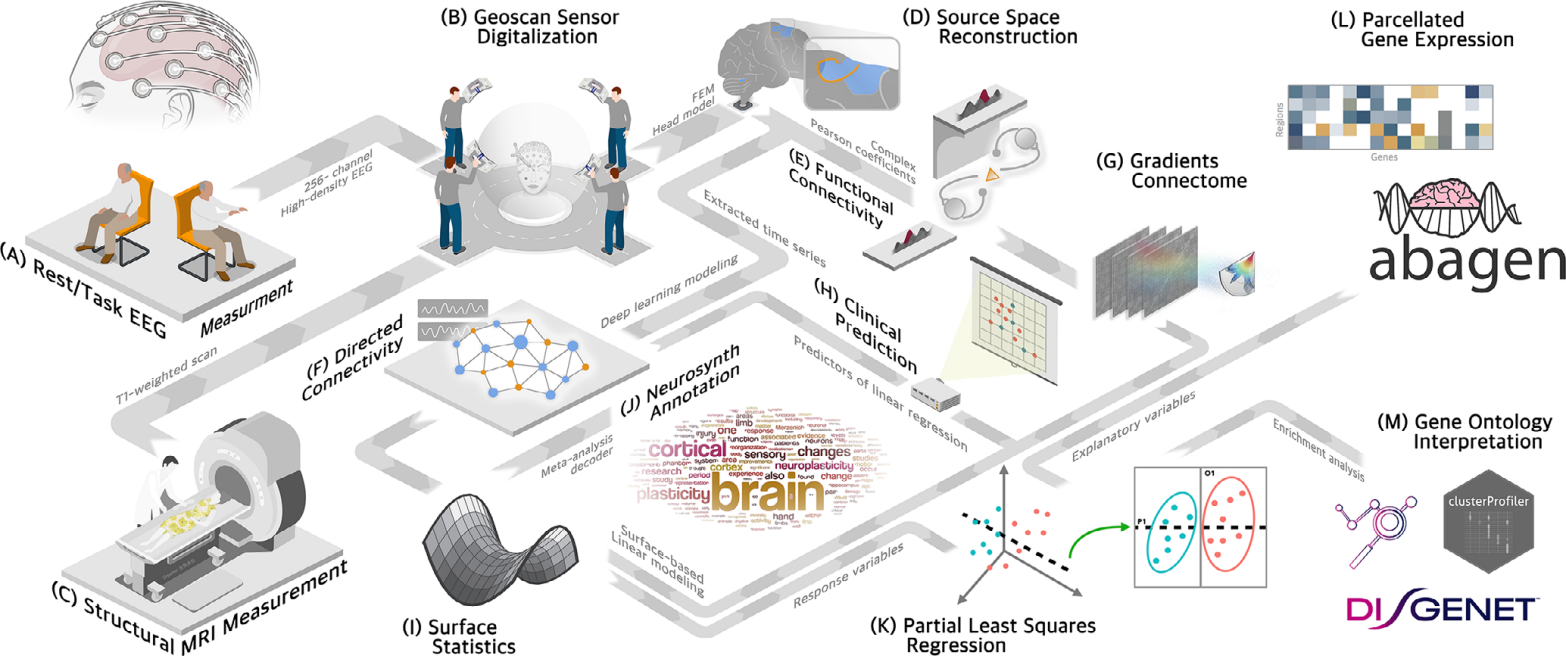
Methodology pipeline. (**A**) High‐density electroencephalography acquisition during rest and motor task. (**B**) Realistic sensor digitalization for precise source reconstruction. (**C**) Magnetic resonance imaging (MRI) acquisition. (**D**) Finite Element Method (FEM) Source reconstruction. (**E**) Functional connectivity computation. (**F**) Directed functional connectivity estimation. (**G**) Principal gradient analysis. (**H**) Prediction analysis for clinical relevance. (**I**) Surface statistics for gradients. (**J**) Neurosynth decoding (**K**) Partial least squares regression. (**L**) Microarray expression extraction. (**M**) Gene enrichment analysis. [Color figure can be viewed at wileyonlinelibrary.com]

## Subjects and Methods

### Study Participants

This study included 35 patients who were clinically diagnosed with idiopathic PD according to the Movement Disorder Society criteria.^18^ The patients had a mean age of 60.80 ± 10.50 years and a mean disease duration of 8.50 ± 6.88 years (Table 1). The general inclusion criteria for patients with PD were: (1) the ability to maintain a forward arm‐holding posture for 2 minutes without noticeable tremor, (2) stable medication use over the past month, and (3) current use of antiparkinsonian medication. The exclusion criteria were a history of other neurological or psychiatric conditions, advanced PD (stage IV or V as determined by the Hoehn and Yahr [H&Y] scale),^19^ or clinical diagnosis of dementia or other clinically significant cognitive impairment. To minimize movement artifacts during electrophysiological recordings, we conducted all sessions in the *on* medication state. Although nine patients were clinically identified as having a tremor‐dominant phenotype, their resting‐state tremor power ratio (3–12 Hz) after medication was not significantly different from the control subjects (*P* > 0.05; see Supporting Information Data S1), confirming effective tremor suppression. A control group of 34 age‐matched volunteers (mean age, 62.03 ± 9.82 years) with normal neurological examinations was also included. Recruitment of patients with PD was conducted by reaching out to both inpatients and outpatients of the Movement Disorder and Neurostimulation Section of the Neurology Department at the University Hospital Mainz (Mainz, Germany). All participants provided written informed consent. The study was approved by the University Hospital Mainz institutional review board and followed the Declaration of Helsinki.

### Clinical Assessments and Experiment Procedures

Clinical motor impairment and disability in PD were assessed using the Unified Parkinson's Disease Rating Scale Part III (UPDRS‐III)^20^ and H&Y^19^ scales by an experienced neurologist. The experiment was conducted in the same environment where the clinical motor examination took place. During the experiment, participants were seated in a comfortable chair with their forearms supported to the wrists by firm armrests. EEG recording began with a 5‐minute resting state, with eyes closed, followed by a 2‐minute hand‐holding condition, where the experimenter instructed the participant to hold both hands outstretched in front of their chest (Fig. 1A), keeping their eyes fixed on a cross point 2 m away. At the end of the EEG experiment, each EEG sensor position was digitized (Fig. 1B) using stereo camera‐tracking technology (GeoScan, MagstimEGI, Roseville, MN, USA).^21^ All participants underwent structural magnetic resonance imaging (MRI) either on the same day (before or after the experiment) or within the same week for the needs of source reconstruction analysis (Fig. 1C).

### Neuroelectrophysiology Acquisition and Processing

A high‐density 256‐channel EEG recording system was employed to collect electrophysiology data with a sampling rate of 1000 Hz. The preprocessing and cleaning procedures were performed in EEGLAB. Preprocessing included the systematic removal of artifact‐prone peripheral channels, retaining 203 reliable sensors.^22^, ^23^, ^24^ The cleaned EEG data were exported for subsequent source analysis. To estimate the source‐level brain activity, we used individual T1‐weighted MRI to build a realistic head model via the finite element method and applied the linearly constrained minimum variance beamformer to minimize spatial leakage and estimate source activity. The estimated source activity was further parcellated into 360 cortical regions based on the Glasser atlas using the scout function of principal component analysis^9^ (detailed in the Supporting Information).

### Functional Gradients and Directional Connectivity Centrality Estimation

To obtain low‐dimensional gradients from high‐dimensional functional connectivity, we computed imaginary Pearson's correlations from source‐space time series filtered into pathophysiologically relevant frequency bands. We prioritized the beta (13–30 Hz) and gamma (60–80 Hz) bands given their established association with motor symptom dynamics and their potential as control signals for neuromodulation. For completeness, exploratory analyses of the alpha band (8–12 Hz) were also conducted and are documented in the Supporting Information. This process yielded a functional connectivity matrix for each frequency band per participant, which we used to compute cortex‐wide functional gradients using BrainSpace (version 0.1.10).^25^ Following an established pipeline (for step‐by‐step breakdown and detailed parameters, see Supporting Information),^17^ we retained the top 10% of weighted, z‐transformed connections, constructed an affinity matrix with a normalized angle kernel, and applied diffusion mapping to identify principal gradients.^17^ Individual gradients were aligned to the control group via Procrustes alignment, and between‐group differences were tested using surface‐based linear models in BrainStat.^26^ Functional interpretation of the gradients map was inferred with Neurosynth decoding,^27^ and network‐level changes were assessed using the Cole‐Anticevic Brain Network Parcellation.^28^

For regions showing gradient alterations, we estimated directed connectivity using the VARDNN toolbox,^29^ decomposing the resulting connectivity matrix into outward and inward flows.^30^ Nodal source and sink degrees were calculated by summing respective inward/outward weights. To understand the relationship between altered functional gradients and directed connectivity, we performed a correlation analysis, providing complementary insights into brain topography.

### Prediction of Clinical Assessment

Further exploration of the clinical relevance involved assessing the diagnostic power for PD by classifying patients with PD and control subjects, as well as evaluating the predictive power for UPDRS‐III scores using the functional gradients, along with their source and sink degrees. We employed a simple linear regression model with least absolute shrinkage and selection operator (LASSO) regularization for the classification and prediction assesments.^31^ We used a nested cross‐validation approach with five inner and outer splits, and this process was repeated 100 times with random shuffling. The distribution of performance metrics was explicitly analyzed to safeguard against overfitting and to ensure an unbiased evaluation of model stability (for a detailed breakdown, see Supporting Information).^32^, ^33^, ^34^

### Disease‐Relation Validation Based on Enrichment Analysis

To validate our findings and assess their relevance to PD, we linked the group‐level T map with the Allen Human Brain Atlas (microarray dataset with 3702 samples from postmortem brain) using partial least squares (PLS) regression.^35^ Bootstrapping (1000 iterations) identified robust gene contributors by calculating *Z*‐scores (bootstrap ratios). Ranked genes then underwent Gene Ontology (GO), DisGeNET, and Kyoto Encyclopedia of Genes and Genomes (KEGG) enrichment analyses (see Supporting Information for details).^36^ Results were false discovery rate (FDR) corrected for multiple comparison.^37^

**TABLE 1:** Study demographics

Characteristics	PD (n = 35)	HC (n = 34)	*P*
Mean age, yr	60.80 (10.50)	62.03 (9.82)	0.6175
Sex, n (%)			0.0384*
Male	26 (74)	16 (47)	
Female	9 (26)	18 (53)	
Disease duration, yr	8.50 (6.88)	N/A	
UPDRS‐III scores	21.61 (9.33)	N/A	
H&Y scale	2.80 (0.86)	N/A	
Handedness (right/left)	34/1	34	1
LEDD, mg	742.86 (516)	N/A	
Tremor dominant, n (%)	9 (26)	N/A	

Abbreviations: PD, Parkinson's disease; HC, healthy control; UPDRS, Unified Parkinson's Disease Rating Scale; H&Y, Hoehn and Yahr scale; LEDD, levodopa equivalent daily dose; N/A, not applicable.

## Results

### Demographics

### Between‐Group Differences in Principal Gradient Analysis

To map the large‐scale organization of the cortex, we applied an established dimensionality reduction approach (diffusion mapping).^17^ We identified the principal cortical gradient, which serves as the primary axis of functional organization (Supporting Information Fig. S3). This principal component provides a stable representation of the cortical connectivity backbone. We subsequently compared this topographical organization between patients with PD and healthy control subjects (HCs) to identify disease‐related disruptions (Supporting Information Fig. S3B). Although we focused on the beta and gamma bands because of their established relevance to motor pathophysiology in PD, alpha‐band analyses were also conducted to provide a comprehensive spectral overview. These results are reported in the Supporting Information (Fig. S4) to maintain the focus on primary motor‐relevant biomarkers.

**FIG. 2:**
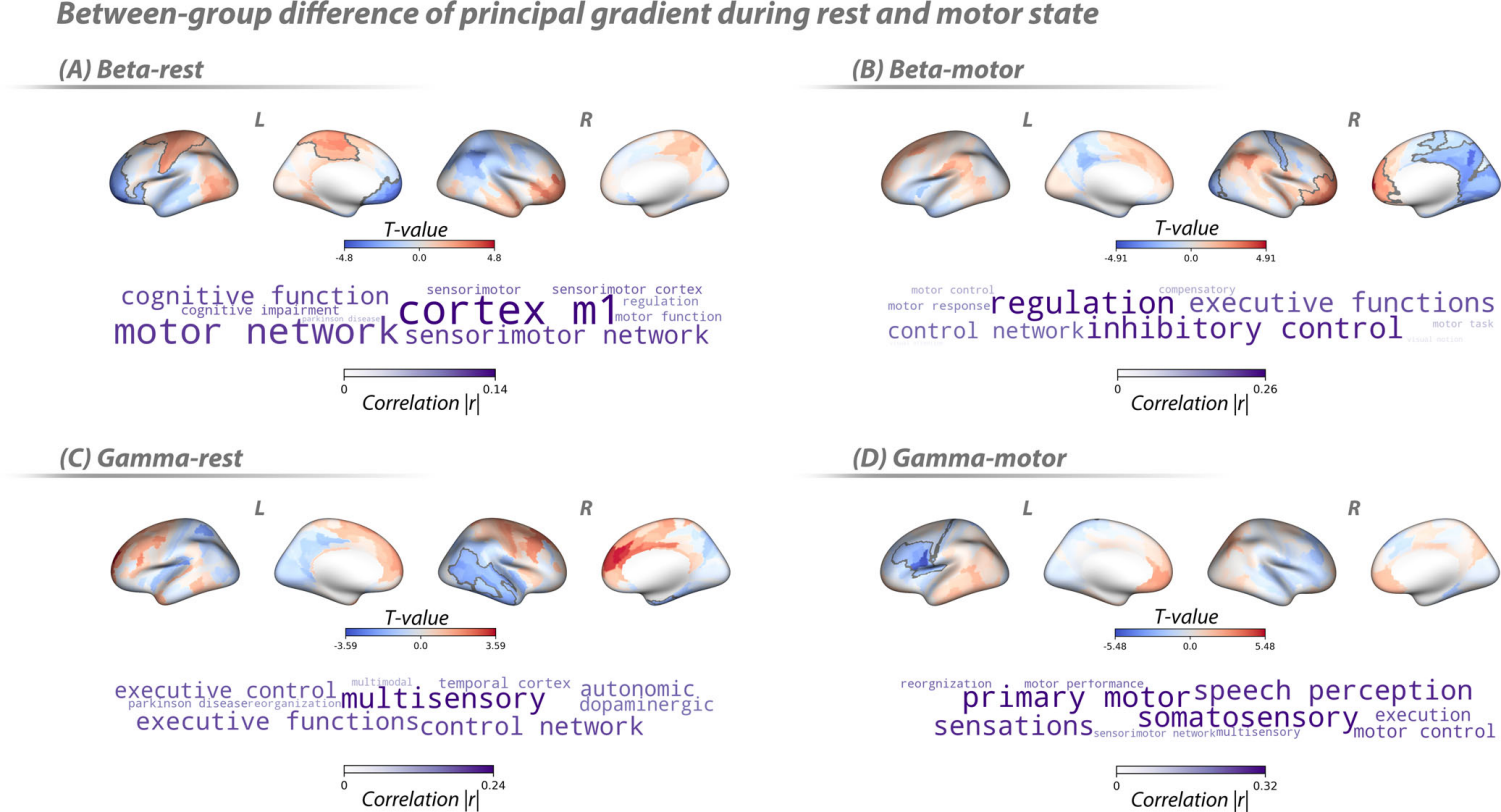
Group contrasts of the principal gradient and associated functional association. Group‐level T‐value maps showing differences in the principal gradient between PD and HC groups (computed as PD minus HC) during (**A**) resting state, beta band; (**B**) motor state, beta band; (**C**) resting state, gamma band; and (**D**) motor state, gamma band. Each panel includes the gradient contrast maps (top) and functional term associations derived from Neurosynth decoding (bottom). HC, healthy control; PD, Parkinson's disease; |r|, absolute Pearson correlation coefficients. [Color figure can be viewed at wileyonlinelibrary.com]

The resting‐state beta gradient exhibited a posterior‐anterior axis (Supporting Information Fig. S3B), with patients with PD showing significantly higher values in unimodal sensory‐motor regions and lower values in transmodal prefrontal and visual areas (*P*_FDR_ < 0.05; Fig. 2A). During the motor state, patients with PD showed higher values in the prefrontal cortex and lower values in visual and motor regions (*P*_FDR_ < 0.05; Fig. 2B). Gamma‐band alterations were noted in the temporal cortex at rest and shifted to the precentral gyrus and motor‐related regions during the task (*P*_FDR_ < 0.05; Fig. 2C,D).

Further, we used the Neurosynth Decoder to assess the spatial correlation between the observed T‐map and meta‐analytic functional maps. The resulting functional profiles (Fig. 2) illustrate the top 10 terms most strongly correlated with the between‐group spatial pattern. During the resting state, beta‐band gradient differences were most strongly associated with motor and transmodal functions, whereas gamma‐band differences were linked to multisensory functions and, notably, to PD itself. During the motor state, beta‐band differences correlated with inhibitory control and executive functions, whereas gamma‐band differences showed prominent associations with somatosensory and motor networks. For a complete list of terms and their correlation values, see the Supporting Information Tables S1–S7. To complement the gradient analysis, we performed gradient stratification by an established functional network (see Supporting Information Figure S5).

### Relation to Information Flow Alterations

**FIG. 3:**
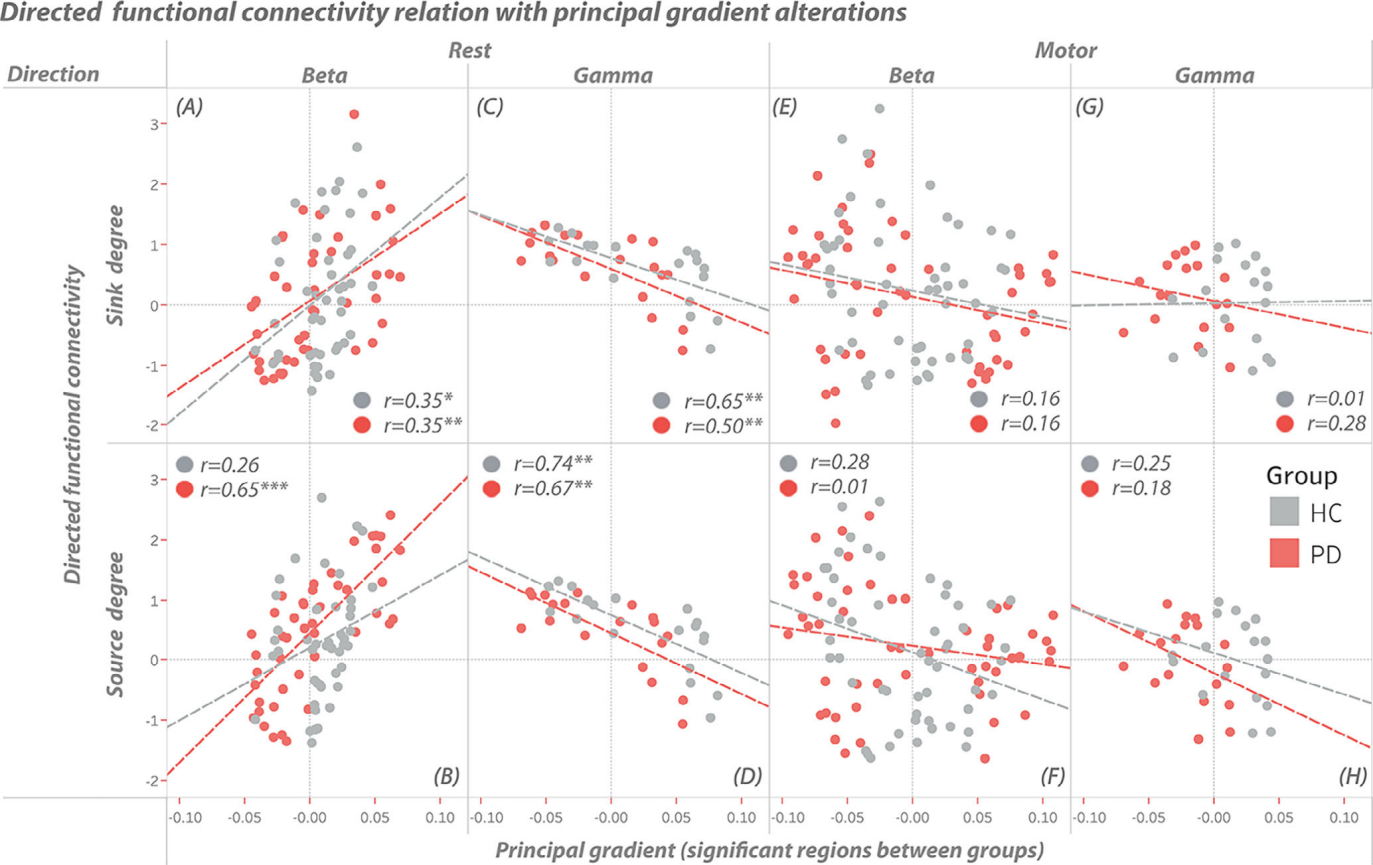
Correlation between regional gradient alterations and corresponding functional information flow. Group‐level averages of altered regional gradient scores were used in correlation analyses with functional information flow, assessed as sink (inward) and source (outward) degrees. The correlation was performed for both resting and motor states, across beta and gamma frequency bands: (**A**–**D**) resting state: beta and gamma bands, sink and source degrees; (**E**–**H**) motor state: beta and gamma bands, sink and source degrees. Asterisks indicate statistical significance after correction for multiple comparisons (*P < 0.05, **P < 0.01, ***P < 0.0001). HC, healthy control; PD Parkinson's disease; *r*, Pearson correlation coefficient. [Color figure can be viewed at wileyonlinelibrary.com]

Building on the between‐group analysis of functional gradients, we examined the relationship between the topographic changes observed in the regions showing group differences and regional information flow measures: overall sink (inward) degree centrality and source (outward) degree centrality as shown in Figure 3.

During the resting state, both patients with PD and HCs exhibited a consistent direction of correlation across frequency bands, within both sink and source degrees (Fig. 3A–D). In the beta frequency band, gradient values demonstrated positive correlations with sink and source degrees in the same regions (Fig. 3A,B). Beta‐band gradient scores correlated positively with sink and source centrality across groups (Fig. 3A,B), with the source degree association being significantly stronger in PD (*r* = 0.65, *P*_FDR_ < 0.001). Gamma‐band gradients showed significant negative correlations with both centrality measures in both groups, although associations were numerically weaker in PD (Fig. 3C,D).

In the motor state, between‐group differences in the principal gradient within both the beta and gamma frequency bands showed negative correlations with sink and source degrees. Even though some associations reached nominal significance (*P* < 0.05), they did not remain significant after correction for multiple comparisons. Nevertheless, observable trends were present, specifically in the beta band with source degree (HC: *r* = 0.28, *P*_FDR_ > 0.05; PD: *r* = 0.01, *P*_FDR_ > 0.05) and in the gamma band with sink degree controls (HC: *r* = 0.01, *P*_FDR_ > 0.05; PD: *r* = 0.28, *P*_FDR_ > 0.05), where moderate correlation coefficients were observed.

### Prediction of Clinical Assessments

**FIG. 4:**
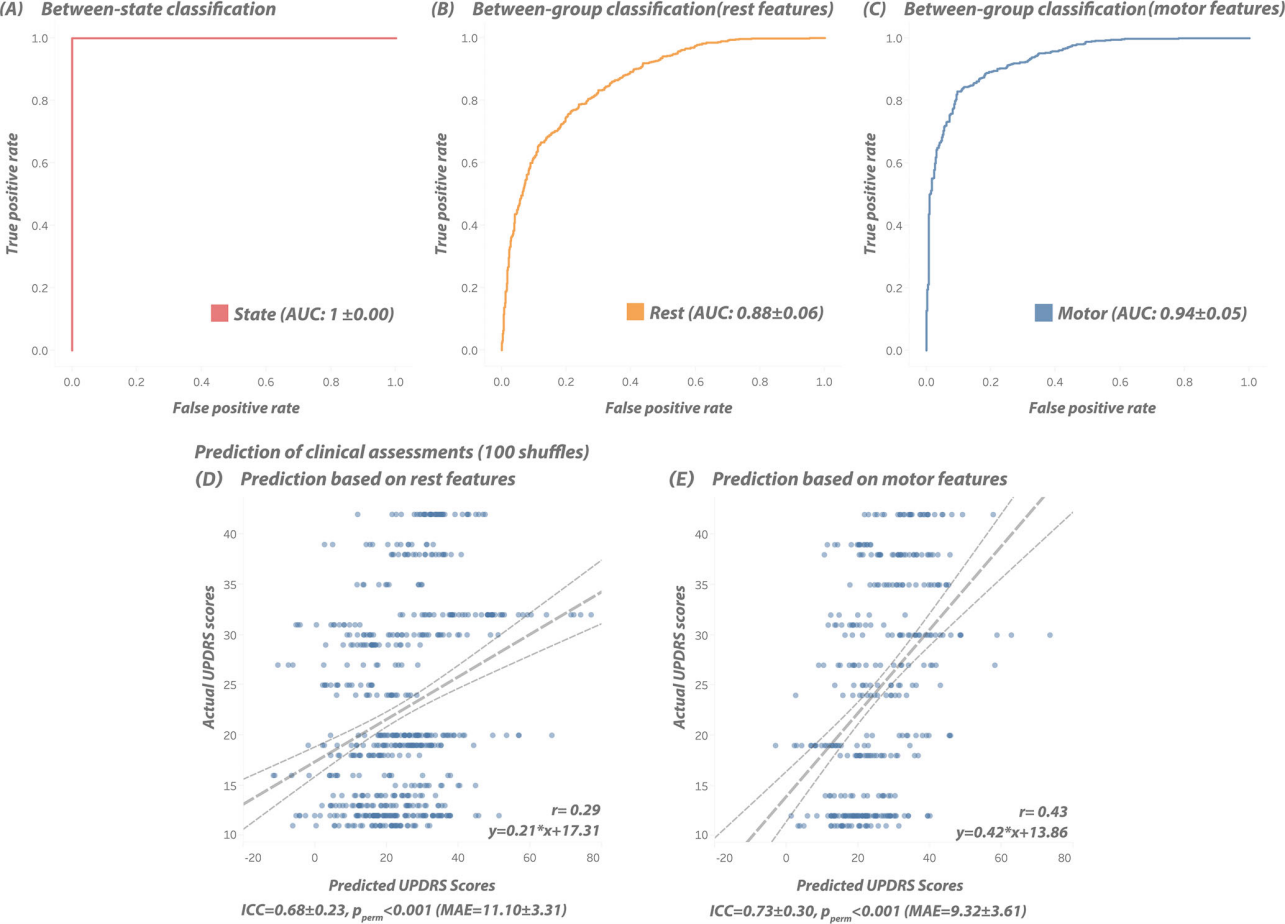
Between‐state and diagnostic classification analysis. Classification was performed using linear regression with LASSO regularization for feature selection, evaluated through a nested cross‐validation framework with 100 repetitions of random shuffling. Input features included principal gradient scores and corresponding regional information flow measures. Each subfigure presents the average AUC. (**A**) State classification (rest vs. motor) in patients with PD, using pooled features from regions showing group‐level alterations across both states. (**B**) Diagnostic classification (PD vs. HC) using resting‐state features. (**C**) Diagnostic classification (PD vs. HC) using motor‐state features. Prediction was performed using linear regression model LASSO regularization for feature selection, evaluated through a nested cross‐validation framework with 100 repetitions of random shuffling. Input features included principal gradient scores and corresponding regional information flow measures. Each subfigure presents the relationship of the actual Unified Parkinson's Disease Rating Scale Part III (UPDRS‐III) score and predicted UPDRS‐III scores. The data distribution across 100 repetitions serves to demonstrate the model robustness against overfitting rather than a single model fit. ICC indicates the consistence and stability between the prediction of each repetition. (**D**) UPDRS‐III prediction using resting‐state features. (**E**) UPDRS‐III prediction using motor state features. AUC, area under the curve; HC, healthy control; LASSO, least absolute shrinkage and selection operator; MAE, mean absolute error; *P*_perm_, statistical *P* value obtained through permutation tests; PD, Parkinson's disease; *r*, Pearson correlation coefficient; UPDRS‐III, Unified Parkinson's Disease Rating Scale; y, fitted linear model equation. [Color figure can be viewed at wileyonlinelibrary.com]

Building on the gradient analyses, we examined the clinical utility of the topographic features that demonstrated between‐group differences in classification and prediction tasks. Specifically, we assessed their ability to distinguish between motor and resting states, differentiate patients with PD from HCs (Fig. 4A–C), and predict clinical motor severity (Fig. 4D,E) as reflected by a higher score on the UPDRS‐III. Constraining the feature space to these significant regions acted as a biological prior to reduce the risk of spurious associations. Classification and regression were both conducted with a linear regression model featuring LASSO regularization, which was employed to prevent overfitting and to filter out redundant regional features. The non‐zero coefficients in LASSO regression, which refer to feature selection, were normalized as selection probability (Supporting Information Figs. S7 and S8). The model was evaluated using a nested cross‐validation framework, and the procedure was repeated 100 times with random shuffling.

Between‐state classification achieved perfect accuracy (area under the curve = 1.00; Fig. 4A), driven by distinct topographical features detailed in the Support Information S7. Whereas resting‐state features significantly predicted UPDRS‐III severity (*r* = 0.29; *P*_perm_ < 0.001; Fig. 4D), motor‐state models demonstrated superior predictive performance (*r* = 0.43; *P*_perm_ < 0.001; Fig. 4E). Both models showed high consistency across cross‐validation folds (intraclass correlation coefficient > 0.68), confirming the robust relationship between topographical features and clinical motor burden.

### Molecular Correlates of Topographic Alterations

To provide biological validation for our findings, we linked group‐level T‐maps with regional gene expression from the Allen Human Brain Atlas. Using PLS regression, we identified and ranked genes whose spatial expression patterns matched our topographic results, determining their enrichment in relevant biological pathways and disease associations (Supporting Information Fig. S6).

GO analysis demonstrated that both frequency bands were associated with fundamental cellular processes, including signaling molecule release, nervous system development, ion transport, and protein secretion (*P*_FDR_ < 0.05). KEGG pathway analysis further identified calcium signaling as a shared molecular feature across both beta and gamma bands (*P*_FDR_ < 0.05). Interestingly, the gamma‐band topography showed a more extensive molecular profile, with specific enrichment in pathways central to PD, such as axon guidance and dopaminergic and glutamatergic synapse function (*P*_FDR_ < 0.05). Finally, disease‐gene network analysis confirmed that these topographic patterns are strongly enriched for parkinsonian disorders, muscle rigidity, and Lewy body disease. These results suggest that the frequency‐specific changes observed in our study are anchored in the known molecular architecture of PD. For the complete statistical results, refer to Supporting Information Tables S4–S7.

## Discussion

This study presents a novel characterization of the large‐scale cortical architecture in PD through the lens of functional gradients. Our findings identify a primary axis of cortical connectivity that serves as a stable hierarchical framework that remains consistent for reliable measurement yet adapts predictably between resting and motor states. This balance of stability and state sensitivity suggests that functional gradients constitute a robust spectral‐topographic fingerprint of the PD cortex.

The clinical utility of this framework is demonstrated by its close alignment with the underlying biological and symptomatic profile of the disease. We found that these frequency‐specific topographic patterns are spatially anchored in the molecular architecture and effectively encode clinical motor severity. By showing that functional gradients offer a reliable and state‐responsive map of pathophysiology, these results establish a foundation for neuromodulation tuned to PD‐specific functional and clinical state.

### Oscillatory Reorganization Along Cortical Gradients

Our methodology captures large‐scale, stable organizational structures of the brain. The resting‐state gradient pattern follows a posterior‐anterior axis, consistent with findings from other neuroimaging modalities and studies on structural features and gene expression.^11^, ^12^, ^13^ The functional gradient is a key organizing principle of the human cortex, and its gradual transition reflects the hierarchy of brain function.^13^ The gradients pattern reported in this work is consistent with prior work by Mahjoory and colleagues,^38^ who observed a similar posterior‐anterior organization during the resting state for the beta band. Our study extends these findings by including higher‐frequency gamma oscillations (60–80 Hz), which serve as a potential control signal for aDBS in PD.^39^ We found that the core architecture of gamma oscillations involves transmodal regions engaged in higher‐order functions, with disease pathology altering this activity in a state‐dependent manner. Our analysis showed a shift in gamma‐band organization from the temporal cortex during the resting state to the prefrontal and motor regions during motor tasks. This redistribution aligns with the neural compensation hypothesis, which proposes that patients with PD increasingly recruit prefrontal regions to support executive control and integration. Such a shift suggests that coordinated interactions between the prefrontal and temporal cortices may serve as a key compensatory mechanism to maintain function despite the disease's impact on primary motor circuits.

Crucially, this macroscale reorganization is intrinsically linked to alterations in regional information flow. By integrating directional centrality measures,^29^ we identified distinct shifts in core motor and regulatory networks during beta oscillations, alongside changes in multisensory networks within the gamma band. The significant correlation between principal gradient scores and beta‐band source degree (Fig. 3B) suggests that the observed topographical shifts reflect a disease‐specific reconfiguration of how neural signals propagate across the cortex. This departure from normative connectivity, potentially anchored in the behavior of traveling waves and counterstream architectures,^40^ likely reflects an imbalance between excitation and inhibition within motor circuits. Although some trends in the motor state did not survive rigorous correction for multiple comparisons, the overall alignment between topography and information flow suggests a functional collapse of the normative cortical hierarchy in PD.

### Evaluating Computational Features for Clinical Utility

Validating computational biomarkers for clinical use requires a balance between accuracy and generalizability. To avoid overfitting in our moderate‐sized cohort, we used a simple, repetitive linear model that prioritizes robust feature selection over maximum predictive accuracy.^41^ This approach demonstrated a clear differentiation between resting and motor states, with classification primarily driven by the precuneus (beta band) and frontal opercular area 4 (gamma band) (Supporting Information Fig. S7). Notably, motor‐state features consistently outperformed resting‐state measurements in predicting both disease instance and clinical severity. Although neuroimaging studies traditionally favors the neutral stability of the resting state,^42^ our results align with emerging evidence that task‐based states such as gait, handwriting, and speech provide superior sensitivity for identifying PD pathology.^43^ We propose that this enhanced performance reflects a task‐unmasking effect, whereby engaging the motor system acts as a physiological stress test, amplifying underlying dysfunctions in the segregation of motor and transmodal hierarchies. Because tremor‐related confounds were rigorously mitigated in our framework, this performance reflects neural pathophysiology rather than movement‐related noise.

By capturing systemic shifts in cortical organization, the gradient framework resolves macroscale pathological changes, which were described as a functional network collapse^44^ that traditional localized features fail to capture (Supporting Information Fig. S2). To address this global severity, we prioritized the total UPDRS‐III score as the clinical target rather than individual subscores (eg, tremor, rigidity). Although distinct symptoms involve unique circuits,^45^, ^46^ composite motor scores serve as the primary benchmark for aDBS efficacy.^47^ Furthermore, MDS‐UPDRS subscores are highly intercorrelated.^48^ In our moderate‐sized cohort, disentangling these collinear factors could lead to false positives. By predicting the total score, we capture the shared variance among signs to effectively estimate the global parkinsonian state.

By establishing these functional gradients as a representative template, this work provides a foundational reference map that can be personalized to track an individual‐specific motor state. Because gradients offer a more stable and biologically grounded window into the patient condition compared with traditional subcortical beta power, which is often highly variable and prone to stimulation artifacts, these features are highly effective for predicting clinical severity. Our findings demonstrate that these features are predictive of clinical severity. Consequently, the predictive validity of this gradient‐based framework positions these biomarkers as promising candidates for control signals in aDBS. Leveraging the high temporal resolution of electrophysiological measures such as EEG or electrocorticography, the feasibility of extracting these features in real‐time further supports their integration into next‐generation closed‐loop systems, potentially enabling more precise and individualized therapy for PD. In summary, this work represents a conceptual shift from viewing cortical electrophysiology as a collection of isolated oscillations toward a holistic, hierarchical framework. This template serves as a guide for identifying optimal sensing sites and directing the next generation of multisite cortical‐guided neuromodulation, providing a mechanistic bridge between macroscale information flow and PD pathology.

### Study Limitations

Although this work provides a novel hierarchical framework for PD, several limitations warrant consideration. The sample size and the exclusive focus on the *on* medication state may limit generalizability across the full pharmacological spectrum of the disease. The decision to record in the *on* state was prioritized to ensure signal integrity by minimizing motor artifacts during high‐density EEG acquisition. Furthermore, the transition from eyes closed rest to eyes open task introduces a visual arousal component to state classification, although primary clinical predictions were derived from single‐state analyses to mitigate this confound. Future investigations should incorporate eyes open condition to further disentangle these distinct physiological components. In addition, although the gene enrichment analysis provides essential biological validation, it should be viewed as a descriptive mapping of vulnerability rather than providing direct mechanistic or causal evidence for the observed electrophysiological changes. Finally, the use of an internal control group template for gradient alignment, rather than a large‐scale independent dataset, should be noted. A comprehensive discussion of these factors, alongside detailed methodological caveats, is provided in the Supporting Information.

### Conclusions

In summary, this study demonstrates that the macroscale reorganization of the cortical hierarchy in PD can be robustly characterized through frequency‐specific functional connectivity. By capturing the transitions between resting and motor states, we show that these gradient patterns provide a stable yet task‐sensitive hierarchical reference that is highly predictive of clinical severity. Our findings further demonstrate that these topographical shifts are specifically associated with PD pathology, offering a distinct profile from nonspecific neurodegenerative processes. By identifying optimal sensing sites across different brain states, this work provides a practical framework to guide the development of next‐generation cortical‐guided aDBS.

## Author Roles

(1) Research project: A. Conception, B. Organization, C. Execution; (2) Analysis: A. Design, B. Execution, C. Review and Critique; (3) Manuscript: A. Writing of the first draft, B. Review and Critique.H.D.: 1A, 1B, 1C, 2A, 2B, 2C, 3A, 3B.K.X.: 2C, 3B.M.B.: 3B.H.K.: 1C. J.B.: 1C.B.N.: 3B.J.V.: 3B. S.G.: 1A, 1B, 3B. M.M.: 1A, 1B, 2C, 3B.

## Financial Disclosures and Conflicts of Interest

The authors declare that they have no competing financial or nonfinancial interests (for the preceding 12 months) that could be construed as influencing the work presented in this manuscript. Author disclosures are available in the Supporting Information.

## Supporting information


**Data S1.** Supporting Information.

## Data Availability

To facilitate collaborative research and provide a common reference for the neuroimaging community, the principal gradient templates (based on EEG beta and gamma frequency bands) for the control group are publicly available on GitHub (https://github.com/kenhding/mginoPD). The data are parcellated according to the Glasser atlas. The computation of the gradient was performed using the open‐source BrainSpace toolbox (https://github.com/MICA-MNI/BrainSpace), and analysis was supported by the BrainStat toolbox (https://github.com/MICA-MNI/BrainStat). Both tools are publicly accessible for further research and reproducibility. The raw dataset for this study is not publicly available because it contains information that could breach research participant privacy/consent but is available from the corresponding authors on reasonable request from qualified researchers, within the limitations of the provided informed consent.
