# Supplementary material for: Macroscale Gradient‐Informed Neural Oscillation Topography in Parkinson's Disease
Source: Mov Disord. 2026 Mar 16;41(6):1434–44. doi: 10.1002/mds.70277 (PMC13307240; doi:10.1002/mds.70277)
Supplement: Supplementary file 1 — Data S1. Supporting Information. [file MDS-41-1434-s001.pdf]

Supporting Information for

Macroscale Gradient-Informed Neural Oscillation Topography in Parkinson's Disease

Hao Ding[1,2], Ke Xie[3], Manuel Bange[4], Hannah Kühe[5], Jenny Blech[5], Bahman Nasseroleslami[2], Jens Volkmann[1], Sergiu Groppa[6], Muthuraman Muthuraman[1,4]

[1]Department of Neurology, University Hospital Würzburg, Würzburg, Germany
[2]Academic Unit of Neurology, Trinity College Dublin, Dublin, Ireland
[3]McConnell Brain Imaging Centre,Montreal Neurological Institute and Hospital, McGill University, Quebec, Canada
[4]Institute of Computer Science, University Augsburg, Augsburg, Germany
[5]Department of Neurology, University Medical Center Mainz, Mainz, Germany
[6]Department of Neurology, Saarland University Hospital, Saarland, Germany

Muthuraman Muthuraman
E-mail: muthuraman.muthuraman@uni-a.de

This PDF file includes:

Figs. S1 to S8
Tables S1 to S7
SI References

Contents

1 Materials and Methods 2
1.1 Study Cohort . . . 2
1.2 EEG acquisition and preprocessing . . . 2
1.3 EMG Analysis Pipeline for Tremor Assessment . . . 3
1.4 MRI acquisition and preprocessing . . . 3
1.5 Source reconstruction and temporal dynamics extraction . . . 3
1.6 Functional gradients analysis . . . 3
1.7 Functional decoding based on Neurosynth . . . 4
1.8 Directionality of connectivity centrality analysis . . . 4
1.9 Prediction of clinical assessment . . . 4
1.10 Disease-relation validation based on enrichment analysis . . . 4

2 Control and Validation Analyses 5
2.1 EMG Assessment . . . 5
2.2 Classification and Prediction based on Band Power . . . 6

3 List of Figures 6
3.1 Group-level functional gradient and global density . . . 6
3.2 Functional network-wise analysis . . . 7
3.3 Gene enrichment analysis . . . 8
3.4 Subareas of Significant Clusters . . . 9
3.5 Neurosynth Decoder Correlations Detail . . . 12
3.6 Prediction analysis feature selection frequency by LASSO regression . . . 12
3.7 GO Statistics . . . 13
3.8 KEGG Pathway Statistics . . . 16

4 Supplementary Note 17
4.1 Interpretative scope of gene enrichment analysis . . . 17

5 Study Limitation 17

1. Materials and Methods

1.1. Study Cohort. This study included thirty-five patients who were clinically diagnosed with idiopathic PD according to the Movement Disorder Society (MDS) criteria. The patients had a mean age of 60.80 ± 10.50 years and a mean disease duration of 8.50 ± 6.88 years. The general inclusion criteria for PD patients were: (a) the ability to maintain a forward arm-holding posture for 2 minutes without noticeable tremor, (b) stable medication use over the past month, and (c) current use of antiparkinsonian medication. The exclusion criteria were a history of other neurological or psychiatric conditions (e.g., major depressive disorder), advanced PD (stage IV or V as determined by the Hoehn and Yahr scale) clinical diagnosis of dementia or other clinically significant cognitive impairment.

1.2. EEG acquisition and preprocessing. A high-density 256-channel EEG recording system was employed to collect electrophysiology data with a sampling rate of 1000 Hz. The initial 256 channels were reduced to 203 by excluding channels from the cheeks and lower neck areas, as these regions often experience inconsistent attachment to the skin across participants and tend to capture more muscle artifacts during the experiment. This downsampling approach, as evaluated in previous study, improves the quality of source space analysis while preserving the accuracy of source imaging results.[1] The preprocessing and cleaning procedures were performed in EEGLAB[2], following an established pipeline for source reconstruction with customized parameters adapt to our study.33 In brief, a non-causal low-pass filter was first applied to the raw signal with a cutoff frequency of 100 Hz, followed by resampling to 200 Hz. Subsequently, a non-causal high-pass filter with a cutoff frequency of 0.5 Hz was applied, followed by a notch filter targeting peak frequencies of 50 Hz and 100 Hz. Bad channels were detected and removed using the clear_rawdata function with the following parameters: 'FlatlineCriterion', 5, 'ChannelCriterion', 0.7, 'LineNoiseCriterion', 4. The consecutive time series were segmented into epochs of 5 seconds each, and noisy segments were detected and removed based on joint probability method. Independent component analysis (ICA) decomposition was performed using the Extended Infomax algorithm.[3;4;5] Bad channels were interpolated, and an average reference was calculated. Independent component removal was performed semi-automatically using CORRMAP function,[5] which finds similar ICs across subjects based on map similarity. First, each component was classified by ICLabel,[6] into the following categories: Brain, Muscle, Eye, Heart, Line Noise, Channel Noise, and Other. Second, artifact components were visually inspected and corrected per subject. Since artifacts such as eye blinks exhibit similar quantitative patterns, we manually selected representative IC components of each category as templates. These templated were then used to calculate the correlation between IC components across all subjects. Correlated IC components were classified as artifacts and removed after visual inspection for each subject. Finally, the cleaned EEG data were exported for subsequent source analysis.

1.3. EMG Analysis Pipeline for Tremor Assessment. To objectively quantify the presence of tremor during the resting state, surface electromyography (EMG) was recorded from a subset of nine tremor-dominant PD patients and comparison controls using eight bipolar EMG channels (four per arm) targeting the major muscle groups of the forearms and upper arms. Raw EMG signals were band-pass filtered between 20 Hz and 100 Hz to remove low-frequency movement artifacts and high-frequency noise, after which the analytic envelope was computed via the Hilbert transform to capture instantaneous muscle power. Spectral power density (PSD) was then estimated using Welch's method (Hamming window, 50% overlap), and tremor-band power was calculated as the integrated power within the 3–12 Hz range, expressed as a proportion of the total spectral power. Group-level comparisons (PD vs. HC) were performed across each of the eight channels using two-tailed Welch's t-tests. No significant difference in tremor power ratio compared to healthy controls (all $p > 0.05$) as shown in Talbe S1

1.4. MRI acquisition and preprocessing. Whole-brain imaging data from all subjects were collected using a 3T Siemens TrioTim MRI scanner equipped with a 32-channel head coil. T1-weighted images were acquired using a 3D spoiled gradient echo sequence with the following parameters: repetition time = 1.9s, echo time = 2.52ms, inversion time = 900ms, flip angle = 9°, field of view = 256 × 256 mm, number of slices = 162, and voxel size = 1 × 1 × 1 mm³. T2-weighted images were acquired using a 3D spin-echo sequence with the following parameters: repetition time = 3.2s, echo time = 402ms, flip angle = 120°, field of view = 256 × 256 mm, number of slices = 162, and voxel size = 1 × 1 × 1 mm³. Anatomical images were normalized to Montreal Neurological Institute (MNI) space using SPM12[7] . In CAT12[8] , the T1-weighted images were segmented into five tissue types: gray matter (GM), white matter (WM), cerebrospinal fluid (CSF), skull, and scalp, using the default segmentation approach. The segmented tissue maps were then used for computing the forward model for source reconstruction.

1.5. Source reconstruction and temporal dynamics extraction. Source reconstruction was performed using the Brainstorm toolbox (version 18-Mar-2024).[9] Head meshing was performed using the Headreco[10] functionality of the SimNIBS 3 toolbox[11] to create a tetrahedral mesh from T1- and T2-weighted structural MRI images. Head modeling (forward problem) was solved using the Finite Element Method (FEM) in the open-source software DUNEuro,[12] which reconstructed the MRI volume source space based on 203 EEG electrode positions derived from the Geoscan system aligned with the individual MRI anatomy. The default FEM conductivity values for the five tissues were set as follows: WM = 0.14, GM = 0.33, CSF = 1.79, skull = 0.008, and scalp = 0.43. The continuous Galerkin solver was used with a source model based on Venant moments of 3, a reference length of 20, a weighting exponent of 1, and a relaxation factor exponent of 6. Mixed moments and restrictions were applied. The source space grid resolution was set to 2 mm, matching a common group template grid. For subjects where forward modeling failed due to mesh holes, the head mesh was recomputed with a finer resolution.[13] Inverse modeling was performed using the linearly constrained minimum variance (LCMV) beamformer to estimate source activity. This method scans all potential brain locations specified in the head model, estimating their respective contributions to sensor data while attenuating the contributions from other brain regions. The outcome measure was the pseudo neural activity index score, analogous to a z-score.[14] For dipole scanning, an unconstrained approach was used, which finds the best-fitting dipole at each time point, optimizing the orientation with respect to the data. The median eigenvalue was selected for data covariance regularization, as recommended by the Brainstorm toolbox. Inverse modeling was performed for each epoch, and source-space time series were extracted using Principal Component Analysis (PCA)[15] based on the Glasser atlas parcellation,[16] a multimodal parcellation comprising 180 homologous parcels per hemisphere.

The use of the high-resolution Glasser atlas (360 ROIs) was essential for our gradient analysis to accurately capture the continuous transitions of functional gradients. While the number of parcels exceeds the number of sensors, our pipeline mitigates oversampling concerns through rigorous channel selection (removing artifact-prone peripheral sensors) and the use of the LCMV beamformer, which acts as a spatial filter to suppress crosstalk and leakage. Furthermore, unlike coarser parcellations (e.g., Desikan-Killiany) which may average out subtle hierarchical shifts, the 360-region parcellation offers the necessary granularity to resolve smooth functional boundaries and preserve individual variance in connectivity topography. While the source reconstruction defined a high-resolution volumetric grid (FEM), the subsequent projection onto the surface-based Glasser atlas ensures that our gradient mapping reflects the complex folding patterns and functional organization of the cortex.

1.6. Functional gradients analysis. To decompose the high-dimensional functional connectivity into a low-dimensional gradient representation, we computed functional connectivity using the imaginary component of complex Pearson's correlations between region pairs. This calculation was based on band-pass filtered (4th-order Butterworth filter) time series (extracted from the source space) in the alpha (8–12 Hz), beta (13–30 Hz), and gamma (60–80 Hz) frequency bands.[17] The use of the imaginary component is particularly advantageous as it effectively mitigates the confounding effects of volume conduction, isolating true phase-based interactions between brain regions and thereby providing a more reliable functional connectivity assessment. This process yielded a 360×360 functional connectome matrix for each participant, which was used to compute cortex-wide functional gradients using BrainSpace (version 0.1.10).[18] Following previous studies,[19;20] we retained the top 10% weighted connections per region after z-transforming the connectivity matrix to focus on the most robust functional connections, minimizing the influence of noise and spurious correlations. Next, we constructed an affinity matrix based on the connectivity matrix using a normalized angle kernel to capture the similarity in connectivity profiles between regions.[21;22] To identify principal gradient components that characterize the fundamental organization of cortical connectivity profiles, we employed a diffusion mapping approach on the affinity matrix. This method non-linearly reduced the high-dimensional connectivity to low-dimensional manifolds.[19;23] Unlike linear methods that merely explain the largest variance, this approach captures the intrinsic geometry of the connectivity, with the principal gradient components representing the continuous state transitions spreading across the network. Finally,

Procrustes analysis was used to align the control group for comparison. Between-group differences in functional gradient values were assessed by comparing PD patients and controls using surface-based linear models implemented in BrainStat (version 0.4.2).[24] Additionally, we stratified the functional gradient findings with respect to twelve macroscale functional networks, as defined by the Cole-Anticevic Brain Network Parcellation (CAB-NP),[25] to probe network-level alterations. In these analyses, age and sex were included as covariates, and a false discovery rate (FDR) procedure was applied to account for multiple comparisons.[26]

1.7. Functional decoding based on Neurosynth. To obtain intuitive insight into functional alterations based on the between-group differences in the cortical gradient maps, we utilized the Neurosynth decoder, a large-scale, automated meta-analytic tool that links brain activation patterns to functional terms derived from published neuroimaging studies.[27] The between-group t-map of the principal gradient was used as input to the decoder, which computes spatial correlations between the input map and term-based meta-analytic maps from the Neurosynth database. The resulting functional terms were then ranked based on the strength of their spatial similarity to the input map. This approach allows us to infer the functional significance of the observed alterations. In this work, we focused on functional and disease-related terms, excluding anatomical, psychiatric, and demographic terms. For detailed methodology, readers are referred to the original publication.[28]

1.8. Directionality of connectivity centrality analysis. Building on the identified regional alterations in the gradients, we further examine the connectivity interactions by quantifying directed functional connectivity and calculating the nodal outward and inward degree centrality.[29] Directional functional connectivity between the significant between-group regions was estimated using the Vector Auto-Regressive Deep Neural Network (VARDNN) toolbox.[30] This approach integrates a vector autoregressive model with deep neural network (DNN) architectures to analyze timeseries data. For each subject, autoregressive model coefficients were estimated using the Vieira-Morf algorithm, and the optimal model order was determined via the Bayesian information criterion.[31] Subsequently, a separate DNN was trained for each node (i.e., the timeseries of a single brain region) to predict its timeseries values based on past observations (defined by model order) from all nodes. The Adam optimizer was employed to prevent overfitting during training.[32] During the training phase, the DNN learned autoregressive dependencies through its hidden units. After the training completion, the model input was perturbed one node at a time and passed through the model to generate new predictions. The prediction error between perturbed and unperturbed inputs reflect the influence of one node on another. To quantify this influence as a directed connectivity index, the Granger causality calculation was employed to illustrate the strength index,[33] which can be referred to as the VARDNN-GC index as shown in Equation 1.

$$\mathcal{F}_{j\to i}^{VARDNN-GC} = \log\left(\frac{\mathrm{var}\left(\boldsymbol{Err}_{i\backslash j}\right)}{\mathrm{var}\left(\boldsymbol{Err}_{i}\right)}\right) \qquad [1]$$

WhereErr represents the residuals of the time series, and var denotes statistical variance. For a given node i, the residual error is computed as the difference between the actual and predicted signals. The VARDNN-GC measure is then defined as the logarithm of the ratio of the variance of these residuals. This ratio quantifies the extent to which the exclusion of node j increases the prediction error for node i, thereby indicating the directional influence of node j on node i. VARDNN demonstrated superior accuracy compared to methods like partial correlation and multivariate Granger causality and remains robust and scalable even as the number of nodes considerably increases, where traditional approaches often struggle with efficiency and accuracy.[30] Following the estimation of directed functional connectivity for each node, the resulting connectivity matrix (360 $\times$ 360) was decomposed into two components: the efferent component (columns), representing source/outward signal flow, and the afferent component (rows), reflecting sink/inward degree. For each node, the source and sink degree were calculated as the sum of the efferent and afferent connectivity weights, respectively.[29] To explore the relationship between altered functional gradients and directed centrality in the same regions, we conducted a correlation analysis between these two variables, providing complementary insights into the brain topography.

1.9. Prediction of clinical assessment. Further exploration of the clinical relevance involved assessing the diagnostic power for PD by classifying PD patients and controls, as well as evaluating the predictive power for UPDRS-III scores using the functional gradients, along with their source and sink degrees. While achieving the highest accuracy is not the primary goal of this study, which is more of an engineering question, we aim to provide finding that is both interpretable and generalizable. For this reason, we employed a simple linear regression model for the classification and prediction task.[34;35], We utilized nested cross-validation with both inner and outer five splits.[36;37;38] Given that the inclusion of multiple features (brain regions) increases the dimensionality of the feature space, it raises the risk of overfitting, particularly when predicting a single clinical variable. To mitigate this, we applied Lasso regularization and selected only the non-zero coefficients for the linear regression model in each fold.[39] The optimal model for each run, determined by the inner-loop cross-validation, was selected based on its best predictive performance on the hold-out test fold. This process was repeated 100 times with random shuffling to reduce permutation bias. The results not only demonstrate the predictive power with clinical assessments but also offer insights into the importance of specific brain regions. This approach provides an intuitive understanding of clinical relevance while circumventing the use of black-box models, which was used in prior studies.[35;40]

1.10. Disease-relation validation based on enrichment analysis. To validate the robustness of our findings and assess their relevance to PD, we employed partial least squares (PLS) regression on the significant brain regions identified from between-group differences (dependent variable) and the microarray dataset from the Allen Human Brain Atlas (independent variable),[41]

which comprise 3,702 distinct samples from six postmortem human brains. The model weights, representing the contribution of each gene, were then used to rank gene lists through bootstrapping method. The resulting ranked gene list was then used for downstream transcriptional and enrichment analyses, including Gene Ontology (GO), disease gene network (DisGeNET), and Kyoto Encyclopedia of Genes and Genomes (KEGG) pathway analyses.[41;42;43;44;45;46;47] In brief, the preprocessing pipeline included several key steps: (i) updating probe-to-gene annotations; (ii) filtering probes based on expression intensity; (iii) selecting representative probes per gene; (iv) aligning tissue samples to brain regions; (v) excluding unmatched samples; (vi) normalizing gene expression across genes and samples; (vii) averaging regional expression values within and across donors. This process yielded a final expression matrix consisting of 300 brain regions by 15,632 genes, which was subsequently used for further analyses. Next, we used PLS regression to relate the gene expression of 15632 genes to the between-group differences of brain patterns (t values of principal gradients and corresponding sink and source degree from 300 brain regions). In the PLS regression model, the z-score normalized gene expression matrix (300 regions$\times$15632 genes) was taken as the independent variable, and the z-score normalized between-group t values patterns was treated as the dependent variable. The PLS components, the linear combination of weighted gene expression values, are ranked by the explained variances between independent and dependent variables. Thus, the first PLS component (PLS1) provides the optimal low-dimensional representation of the covariance of the high-dimensional data matrices.[48] We used a permutation test (i.e., n=10,000) to examine whether the explained variance of the PLS component was significantly greater than expected by chance.[46] Moreover, a bootstrapping method was conducted to evaluate the significance of genes contributing to components. Gene weights that fell below the 0.025 percentile and above the 0.975 percentile of the null distribution were classified as significant contributors to PLS1 at an alpha of 0.05 and selected for further analysis. To test whether these ranked significant genes were enriched for genes implicated in the pathogenesis of PD, we used the clusterProfiler package to perform enrichment analyses.[49] Specifically, GO analysis was carried out to assess overrepresented biological processes. KEGG pathway analysis embedded in the package was used to identify relevant signaling and metabolic pathways. In addition, DisGeNET-based enrichment was performed to explore associations with known disease-related gene sets, particularly those linked to neurodegenerative conditions. For a detailed methodological description, we refer the reader to the original method paper. All enrichment analyses results were corrected for multiple comparisons using the FDR correction.[26]

2. Control and Validation Analyses

2.1. EMG Assessment. To objectively quantify the presence of tremor during the resting state, surface electromyography (EMG) was recorded from a subset of nine tremor-dominant PD patients and comparison controls using eight bipolar EMG channels (four per arm) targeting the major muscle groups of the forearms and upper arms (Figure S1). Raw EMG signals were band-pass filtered between 20 Hz and 100 Hz to remove low-frequency movement artifacts and high-frequency noise, after which the analytic envelope was computed via the Hilbert transform to capture instantaneous muscle power. Spectral power density (PSD) was then estimated using Welch's method (Hamming window, 50% overlap), and tremor-band power was calculated as the integrated power within the 3–12 Hz range, expressed as a proportion of the total spectral power. Group-level comparisons (PD vs. HC) were performed across each of the eight channels using two-tailed Welch's t-tests. No significant difference in tremor power ratio compared to healthy controls (all $p > 0.05$) as shown in Table S1.

Channel Index	Mean TPR PD (n=35)	Mean TPR HC (n=34)	p-value
EMG 1	0.70831	0.64926	0.94357
EMG 2	0.74064	0.4468	0.2777
EMG 3	0.44476	0.59733	0.48783
EMG 4	0.81282	0.64344	0.63731
EMG 5	0.4399	0.45105	0.54504
EMG 6	0.63521	0.51843	0.60386
EMG 7	0.52669	0.52362	0.48597
EMG 8	0.79317	0.48232	0.54214

Table S1. Tremor Power Ratio (3-12Hz) Comparison during Resting-state in PD and Healthy Controls

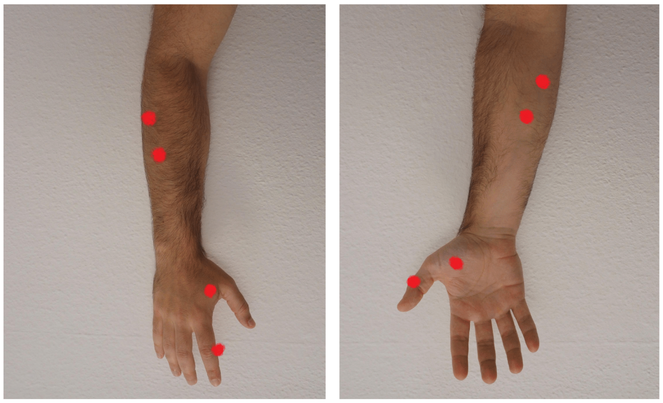

Fig. S1. Surface EMG position

2.2. Classification and Prediction based on Band Power. To validate the information gain of the functional gradient framework, we performed the predictive performance of traditional regional bandpower features (beta and gamma) for group classification and estimating clinical motor severity (UPDRS-III) as shown in Figure S2. The classification analysis revealed that band-power-based predictors were consistently outperformed by functional gradients across all tasks. Interestingly, both motor and resting-state features followed a similar trend: motor-related features yielded higher classification accuracy than resting-state features. This suggests that both local (power) and global (gradient) changes occur between states. However, between-state classification using pooled features failed to match the performance of gradients, further highlighting the superior sensitivity of gradient measures. In the prediction analysis, resting-state features demonstrated statistically significant predictive power for UPDRS-III scores ($r = 0.23$, $p<0.01$confirmed via permutation tests) yet this remained inferior to the performance of functional gradients ($r = 0.29$). In addition, while they showed a moderate correlation ($r = 0.26$) across all the random shuffling, they failed the permutation tests ($p>0.05$) and were significantly outperformed by the gradient measure ($r = 0.43$, $p<0.001$).

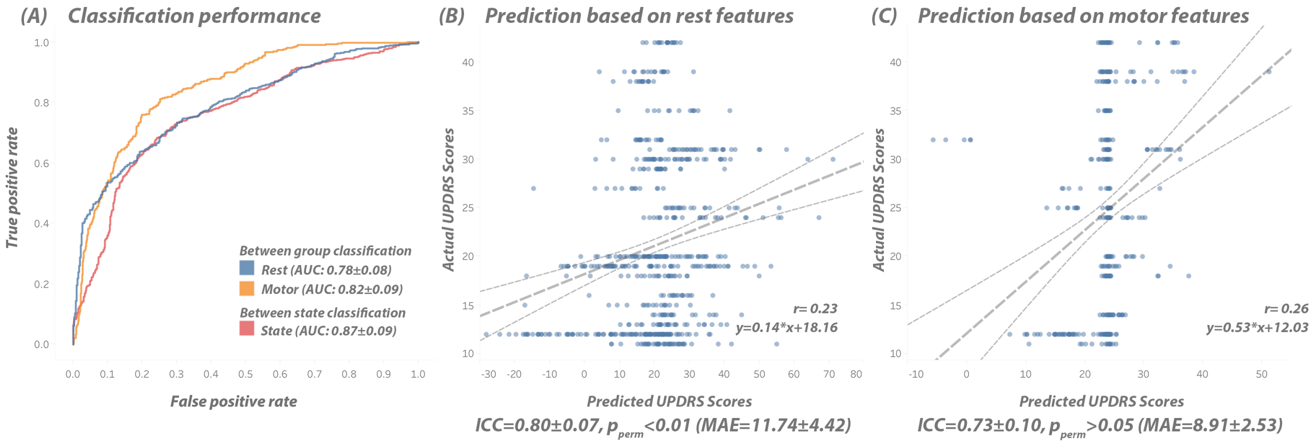

Fig. S2. Between-group Classification and Prediction performance based on band power features.

3. List of Figures

3.1. Group-level functional gradient and global density. In the resting state, the group-level principal gradient was associated with mean eigenvalue of 0.23 in the beta band (PD: 0.23; HC: 0.22; spatial map similarity: rho=0.70, $P_{spin} < 0.001$) and 0.23 in the gamma band (PD: 0.21; HC: 0.26; spatial map similarity: rho=0.74, $P_{spin} < 0.001$). In the motor state, the principal gradient associated with mean eigenvalue of 0.21 in the beta band (PD: 0.22; HC: 0.20; %, spatial map similarity: rho=0.60, $P_{spin} < 0.01$) and 0.21 in the gamma band (PD: 0.20; HC: 0.22; spatial map similarity: rho=0.78, $P_{spin} < 0.01$). These results indicate stable and persistent structures captured by the dimensionality reduction process, with consistent alignment between groups for comparison. In the beta band, the resting-state gradient exhibited a posterior-anterior axis (Figure 2B). Compared to controls, PD patients showed higher gradient values in lateral sensory and motor areas, and lower values in transmodal areas including prefrontal cortex and visual cortex ($P_{FDR} < 0.05$). The gradient distribution in the PD group also showed a broader density spread (Figure S1C). In the motor state, positive gradients were prominent in unimodal regions, with PD patients showing higher values in the prefrontal cortex and lower values in the visual and motor cortices ($P_{FDR} < 0.05$).

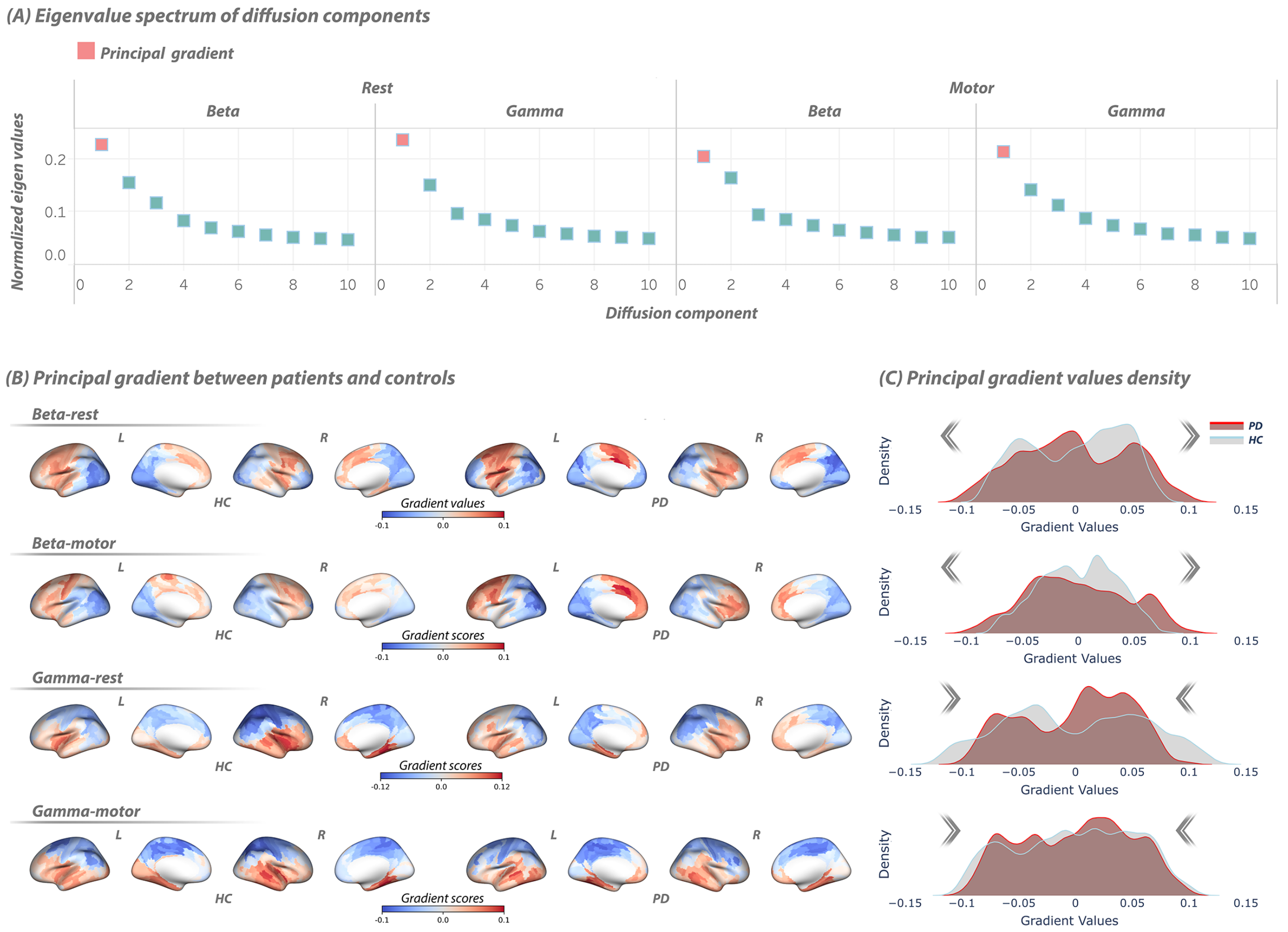

Fig. S3. (A) Diffusion embedding components, with the principal gradient selected based on the highest eigenvalue, capturing the stable structure in high-dimensional functional connectivity. (B) Cortical projection of the principal gradient PD and HC group. (C) Global distribution (whole brain) of the principal gradient values in PD and HC groups. The anchor point indicates the direction of shift (shrinkage or expansion) in the PD group relative to HC. PD Parkinson's disease, HC healthy control.

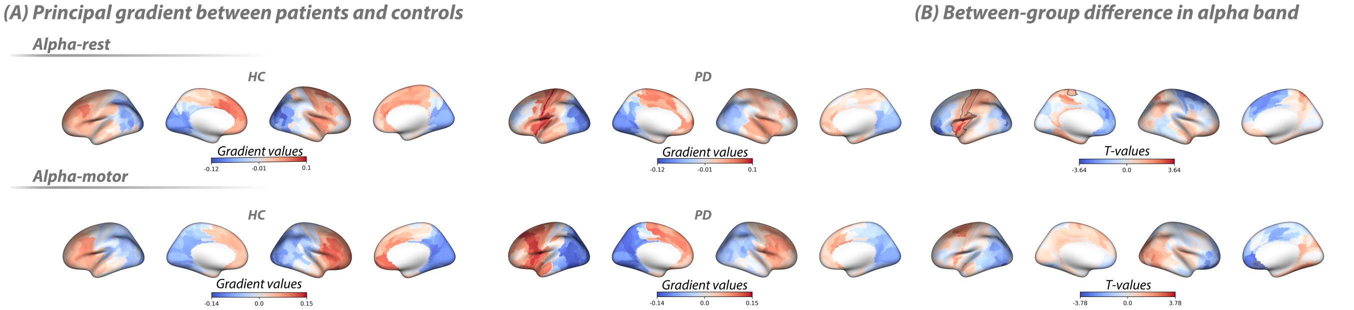

Fig. S4. (A) Cortical projection of the principal gradient PD and HC group in the alpha band (B) Group-level T-value maps showing differences in the principal gradient between PD and HC (computed as PD minus HC) during rest and motor states. PD group relative to HC. PD Parkinson's disease, HC healthy control.

3.2. Functional network-wise analysis. Gradient stratification based on a well-established functional network parcellation revealed distinct network-level alterations associated with beta and gamma frequency bands across both resting and motor states (Figure S2).

Two functional networks in the PD group showed significant state- and frequency-dependent differences in gradient values relative to the HC group. The somatomotor network showed significantly higher gradient scores across both frequency bands during the resting state (beta: t=2.48; gamma: t=2.42; $P_{FDR} < 0.05$). Meanwhile, the ventral multimodal network displayed a significant shift in gradient values, with lower scores in the beta band (t=-2.42; $P_{FDR} < 0.05$) and higher scores in the gamma band (t=3.67; $P_{FDR} < 0.01$) during the motor state compared to the control group. In terms of frequency-related changes, the PD group exhibited lower gradient values in the posterior multimodal network in the beta frequency band (t=-2.88; $P_{FDR} < 0.05$), and higher gradient values in the cingulo-opercular network (t=3.34; $P_{FDR} < 0.01$) during the resting state,

compared to the control group. Additionally, a reduction in gradient values was observed in the primary visual network in the gamma frequency band (t=-2.42; $P_{FDR} < 0.05$). In the motor state, the PD group showed lower gradient values in the primary visual cortex (t=-4.00; $P_{FDR} < 0.01$), along with higher gradient values in the frontoparietal network in the beta frequency band (t=2.92; $P_{FDR} < 0.01$), and higher gradients in the posterior multimodal network in the gamma frequency band (t=3.38; $P_{FDR} < 0.01$), when compared to the control group.

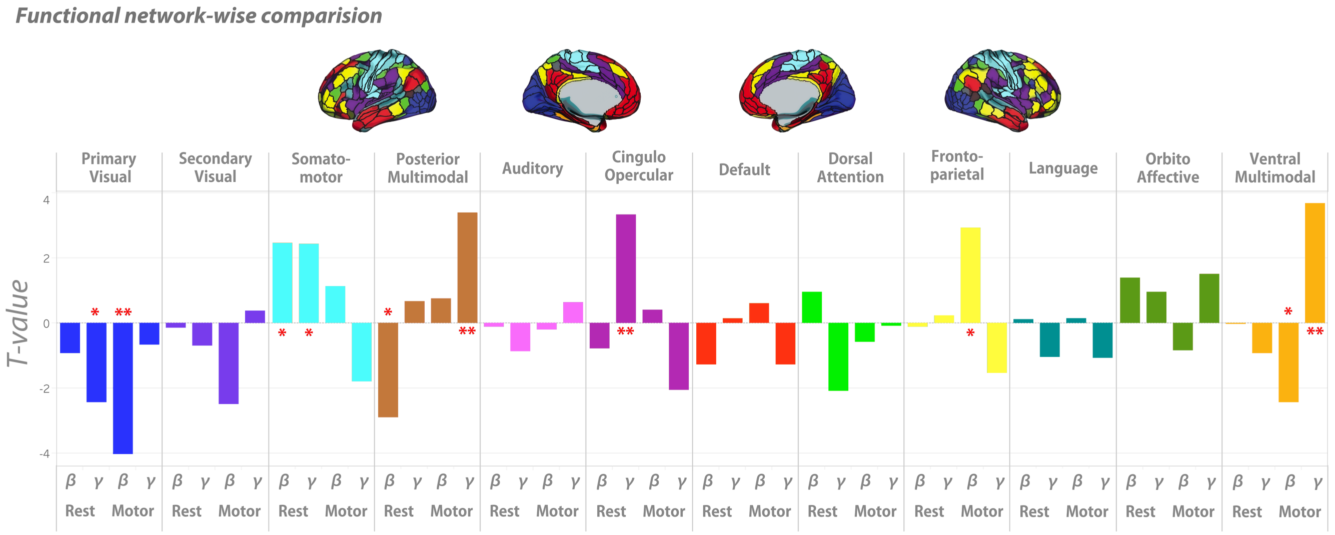

Fig. S5. Functional networks were defined using the Cole–Anticevic Brain-wide Network Parcellation, dividing the cortex into 12 communities (shown along the top axis). Bar plots display T-values representing group differences (PD minus HC) in principal gradient scores. Red asterisks indicate statistical significance after correction for multiple comparisons ($*p$ < 0.05, $**p$ < 0.01). β beta frequency oscillation; γ gamma frequency oscillation. PD Parkinson's disease, HC healthy control.

3.3. Gene enrichment analysis. To evaluate the extent to which alterations in identified topographic features reflect underlying disease-related biological processes, partial least squares (PLS) regression was performed using between-group differences (t-maps) and gene expression data from six postmortem human brains (Allen Human Brain Atlas), matched to the same brain parcellations. The principal PLS component (PLS1) that explains the largest gene–topographic features association, was used to rank gene contributions via bootstrap resampling for enrichment analysis (detailed in Methods and Materials). Figure S3 Gene ontology enrichment analysis identified biological processes including signal release (secretion or discharge of signaling molecules into the extracellular medium), regulation of nervous system development (modulation of the frequency, rate, or extent of nervous system maturation), ion transport (directed movement of ions, such as calcium, across membranes or between cells), protein secretion (controlled release of proteins from a cell), and hormone-related pathways ($P_{FDR} < 0.05$, Figure S3A). KEGG pathway analysis (Figure S3B) revealed a shared calcium signaling pathway associated with both beta and gamma bands ($P_{FDR} < 0.05$). However, gamma topographic features were found to be statistically overrepresented in additional KEGG pathways, including axon guidance, dopaminergic synapse, glutamatergic synapse, and mineral absorption ($P_{FDR} < 0.05$). Furthermore, disease-gene network analysis revealed that the observed differences between beta and gamma topographic patterns were enriched not only in Parkinsonian disorders but also in muscle rigidity, Lewy body disease, sleep disturbances, and central nervous system disorders (Figure S3C). In this section, the most statistically significant terms based on overrepresentation analysis are present. For the complete set of statistical results, refer to the Supplementary Materials Table S3-6.

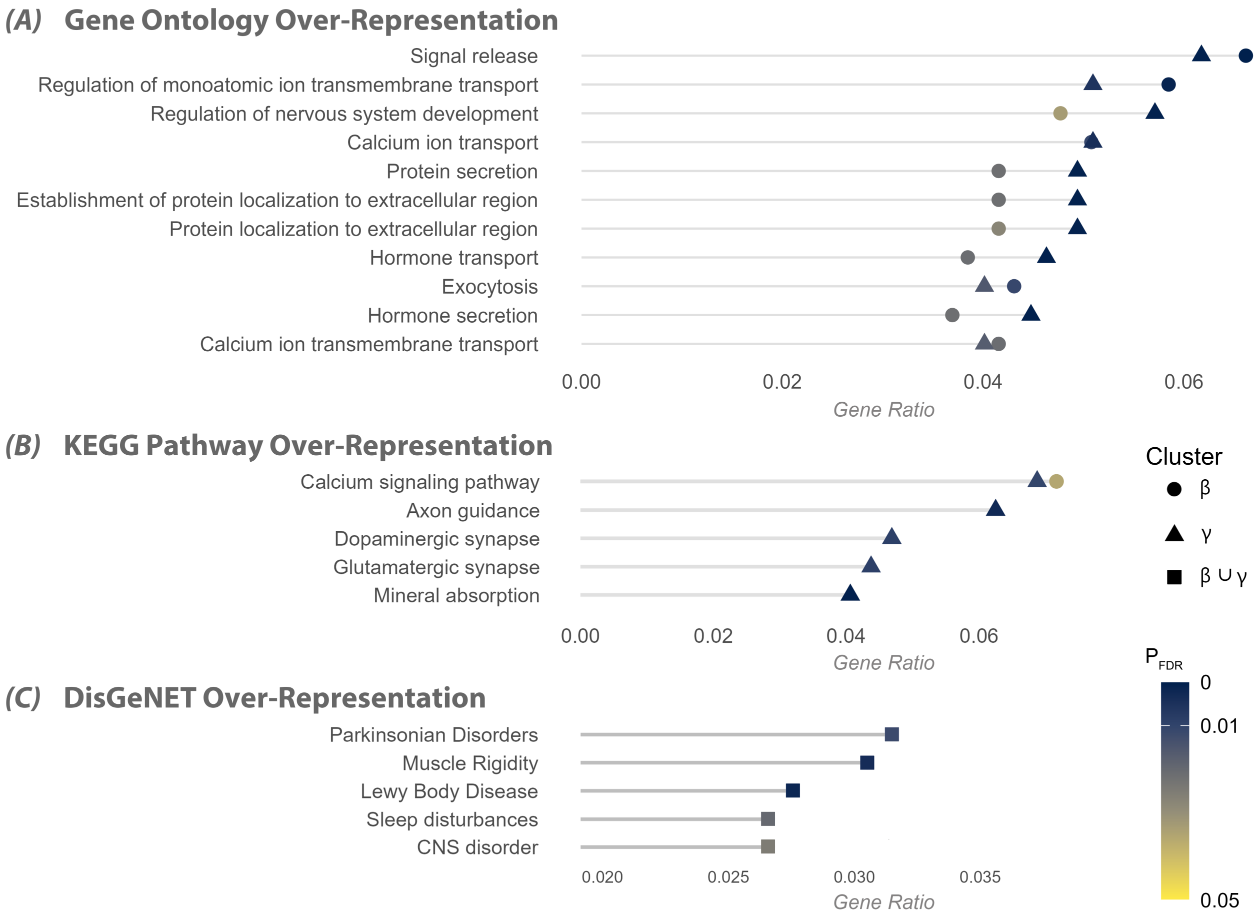

Fig. S6. Enrichment analysis was conducted using three approaches: (A) Gene ontology biological processes, (B) KEGG pathway analysis, and (C) Disease gene network analysis. Statistically significant enriched terms identified by adjusted p-values through FDR are shown in each subfigure for the respective analysis. GO: Gene Ontology; KEGG: Kyoto Encyclopedia of Genes and Genomes; DisGeNET: Disease Gene Network. β: beta frequency oscillation; γ: gamma frequency oscillation; $\beta \cup \gamma$: overlapping genes between the ranked gene lists based on beta and gamma frequency oscillations in the feature space. FDR: False Discovery Rate.

278
3.4. Subareas of Significant Clusters.

Table S2. Statistical significant surface areas based on group comparison of principal gradient using surface linear model

Condition	Frequency	Hemisphere	p Value	Area Name	Area Description
Rest	Beta	Left	0.028	4	Primary Motor Cortex
Rest	Beta	Left	0.028	3b	Primary Sensory Cortex
Rest	Beta	Left	0.028	23d	Area 23d
Rest	Beta	Left	0.028	5m	Area 5m
Rest	Beta	Left	0.028	5mv	Area 5m ventral
Rest	Beta	Left	0.028	23c	Area 23c
Rest	Beta	Left	0.028	5L	Area 5L
Rest	Beta	Left	0.028	24dd	Dorsal Area 24d
Rest	Beta	Left	0.028	24dv	Ventral Area 24d
Rest	Beta	Left	0.028	7AL	Lateral Area 7A
Rest	Beta	Left	0.028	6ma	Area 6m anterior
Rest	Beta	Left	0.028	7Am	Medial Area 7A
Rest	Beta	Left	0.028	7PC	Area 7PC
Rest	Beta	Left	0.028	1	Area 1
Rest	Beta	Left	0.028	2	Area 2
Rest	Beta	Left	0.028	3a	Area 3a

Continued on next page

Table S2 – Continued from previous page

Condition	Frequency	Hemisphere	p Value	Area Name	Area Description
Rest	Beta	Left	0.028	6d	Dorsal area 6
Rest	Beta	Left	0.028	6mp	Area 6mp
Rest	Beta	Left	0.028	p24pr	Area Posterior 24 prime
Rest	Beta	Left	0.028	a24	Area a24
Rest	Beta	Left	0.028	p32	Area p32
Rest	Beta	Left	0.028	10r	Area 10r
Rest	Beta	Left	0.028	47m	Area 47m
Rest	Beta	Left	0.028	9p	Area 9 Posterior
Rest	Beta	Left	0.028	10d	Area 10d
Rest	Beta	Left	0.028	a47r	Area anterior 47r
Rest	Beta	Left	0.028	a9-46v	Area anterior 9-46v
Rest	Beta	Left	0.028	9-46d	Area 9-46d
Rest	Beta	Left	0.028	9a	Area 9 anterior
Rest	Beta	Left	0.028	10v	Area 10v
Rest	Beta	Left	0.028	a10p	Area anterior 10p
Rest	Beta	Left	0.028	10pp	Polar 10p
Rest	Beta	Left	0.028	11l	Area 11l
Rest	Beta	Left	0.028	13l	Area 13l
Rest	Beta	Left	0.028	OFC	Orbital Frontal Complex
Rest	Beta	Left	0.028	47s	Area 47s
Rest	Beta	Left	0.028	6a	Area 6 anterior
Rest	Beta	Left	0.028	AVI	Anterior Ventral Insular Area
Rest	Beta	Left	0.028	31a	Area 31a
Rest	Beta	Left	0.028	25	Area 25
Rest	Beta	Left	0.028	s32	Area s32
Rest	Beta	Left	0.028	pOFC	posterior OFC Complex
Rest	Beta	Left	0.028	FOP5	Area Frontal Opercular 5
Rest	Beta	Left	0.028	p10p	Area posterior 10p
Rest	Beta	Left	0.028	p47r	Area posterior 47r
Motor	Beta	Right	0.003	V1	Primary Visual Cortex
Motor	Beta	Right	0.003	V2	Second Visual Area
Motor	Beta	Right	0.003	V3	Third Visual Area
Motor	Beta	Right	0.003	V4	Fourth Visual Area
Motor	Beta	Right	0.003	V8	Eighth Visual Area
Motor	Beta	Right	0.003	4	Primary Motor Cortex
Motor	Beta	Right	0.003	RSC	RetroSplenial Complex
Motor	Beta	Right	0.003	POS2	Parieto-Occipital Sulcus Area 2
Motor	Beta	Right	0.003	FFC	Fusiform Face Complex
Motor	Beta	Right	0.003	LO1	Area Lateral Occipital 1
Motor	Beta	Right	0.003	LO2	Area Lateral Occipital 2
Motor	Beta	Right	0.003	PCV	PreCuneus Visual Area
Motor	Beta	Right	0.003	7Pm	Medial Area 7P
Motor	Beta	Right	0.003	7m	Area 7m
Motor	Beta	Right	0.003	POS1	Parieto-Occipital Sulcus Area 1
Motor	Beta	Right	0.003	23d	Area 23d
Motor	Beta	Right	0.003	v23ab	Area ventral 23 a+b
Motor	Beta	Right	0.003	d23ab	Area dorsal 23 a+b
Motor	Beta	Right	0.003	31pv	Area 31p ventral
Motor	Beta	Right	0.003	23c	Area 23c
Motor	Beta	Right	0.003	24dd	Dorsal Area 24d
Motor	Beta	Right	0.003	3a	Area 3a
Motor	Beta	Right	0.003	p24pr	Area Posterior 24 prime
Motor	Beta	Right	0.046	a24	Area a24
Motor	Beta	Right	0.046	p32	Area p32
Motor	Beta	Right	0.046	10r	Area 10r

Continued on next page

Table S2 – Continued from previous page

Condition	Frequency	Hemisphere	p Value	Area Name	Area Description
Motor	Beta	Right	0.046	47m	Area 47m
Motor	Beta	Right	0.046	9m	Area 9 Middle
Motor	Beta	Right	0.046	9p	Area 9 Posterior
Motor	Beta	Right	0.046	10d	Area 10d
Motor	Beta	Right	0.046	47l	Area 47l (47 lateral)
Motor	Beta	Right	0.046	a47r	Area anterior 47r
Motor	Beta	Right	0.046	IFSa	Area IFSa
Motor	Beta	Right	0.046	a9-46v	Area anterior 9-46v
Motor	Beta	Right	0.046	9a	Area 9 anterior
Motor	Beta	Right	0.046	10v	Area 10v
Motor	Beta	Right	0.046	a10p	Area anterior 10p
Motor	Beta	Right	0.046	10pp	Polar 10p
Motor	Beta	Right	0.046	11l	Area 11l
Motor	Beta	Right	0.046	13l	Area 13l
Motor	Beta	Right	0.046	47s	Area 47s
Motor	Beta	Right	0.046	AVI	Anterior Ventral Insular Area
Motor	Beta	Right	0.003	ProS	ProStriate Area
Motor	Beta	Right	0.003	VMV1	VentroMedial Visual Area 1
Motor	Beta	Right	0.003	VMV3	VentroMedial Visual Area 3
Motor	Beta	Right	0.003	V3CD	Area V3CD
Motor	Beta	Right	0.003	VMV2	VentroMedial Visual Area 2
Motor	Beta	Right	0.003	31pd	Area 31pd
Motor	Beta	Right	0.003	31a	Area 31a
Motor	Beta	Right	0.003	VVC	Ventral Visual Complex
Motor	Beta	Right	0.046	p10p	Area posterior 10p
Motor	Beta	Right	0.046	p47r	Area posterior 47r
Motor	Gamma	Right	0.031	MST	Medial Superior Temporal Area
Motor	Gamma	Right	0.031	MT	Middle Temporal Area
Motor	Gamma	Right	0.031	PeEc	Perirhinal Ectorhinal Cortex
Motor	Gamma	Right	0.031	A5	Auditory 5 Complex
Motor	Gamma	Right	0.031	STSdp	Area STSd posterior
Motor	Gamma	Right	0.031	STSvp	Area STSv posterior
Motor	Gamma	Right	0.031	TE1a	Area TE1 anterior
Motor	Gamma	Right	0.031	TE2a	Area TE2 anterior
Motor	Gamma	Right	0.031	TF	Area TF
Motor	Gamma	Right	0.031	TE2p	Area TE2 posterior
Motor	Gamma	Right	0.031	PHT	Area PHT
Motor	Gamma	Right	0.031	TPOJ1	TemporoParietoOcci pital Junction 1
Motor	Gamma	Right	0.031	TPOJ2	TemporoParietoOcci pital Junction 2
Motor	Gamma	Right	0.031	TPOJ3	TemporoParietoOcci pital Junction 3
Motor	Gamma	Right	0.031	PGi	Area PGi
Motor	Gamma	Right	0.031	FST	Area FST
Motor	Gamma	Right	0.031	LO3	Area Lateral Occipital 3
Motor	Gamma	Right	0.031	A4	Auditory 4 Complex
Motor	Gamma	Right	0.031	STSva	Area STSv anterior
Motor	Gamma	Left	0.031	PEF	Premotor Eye Field
Motor	Gamma	Left	0.031	3a	Area 3a
Motor	Gamma	Left	0.031	6v	Ventral Area 6
Motor	Gamma	Left	0.031	8C	Area 8C
Motor	Gamma	Left	0.031	44	Area 44
Motor	Gamma	Left	0.031	6r	Rostral Area 6
Motor	Gamma	Left	0.031	IFJa	Area IFJa
Motor	Gamma	Left	0.031	IFJp	Area IFJp
Motor	Gamma	Left	0.031	IFSp	Area IFSp
Motor	Gamma	Left	0.031	p9- 46v	Area posterior 9-46v

Continued on next page

Table S2 – Continued from previous page

Condition	Frequency	Hemisphere	p Value	Area Name	Area Description
Motor	Gamma	Left	0.031	46	Area 46
Motor	Gamma	Left	0.031	43	Area 43
Motor	Gamma	Left	0.031	OP2-3	Area OP2-3/VS
Motor	Gamma	Left	0.031	FOP4	Frontal OPercular Area 4
Motor	Gamma	Left	0.031	FOP1	Frontal OPercular Area 1
Motor	Gamma	Left	0.031	FOP3	Frontal OPercular Area 3
Motor	Gamma	Left	0.031	FOP2	Frontal OPercular Area 2
Motor	Gamma	Left	0.031	Ig	Insular Granular Complex

3.5. Neurosynth Decoder Correlations Detail.

Table S3. the top-ranking relevant terms identified through both positive and negative correlations with the unthresholded t-map

State	Frequency	Term	Correlation
Rest	Beta	cortex m1	-0.14134
Rest	Beta	motor network	-0.1161
Rest	Beta	sensorimotor network	-0.11566
Rest	Beta	sensorimotor	-0.1048
Rest	Beta	sensorimotor cortex	-0.10377
Rest	Beta	motor function	-0.09482
Rest	Beta	parkinson disease	-0.06433
Rest	Beta	cognitive function	0.11069
Rest	Beta	cognitive impairment	0.105319
Rest	Beta	regulation	0.088656
Rest	Gamma	multisensory	0.236979
Rest	Gamma	temporal cortex	0.153747
Rest	Gamma	multimodal	0.110756
Rest	Gamma	autonomic	-0.16685
Rest	Gamma	control network	-0.18766
Rest	Gamma	executive functions	-0.18323
Rest	Gamma	executive control	-0.176
Rest	Gamma	dopaminergic	-0.1566
Rest	Gamma	parkinson disease	-0.15229
Rest	Gamma	reorganization	-0.12846
Motor	Beta	visual attention	0.031377
Motor	Beta	visual motion	0.050418
Motor	Beta	regulation	-0.25645
Motor	Beta	inhibitory control	-0.23208
Motor	Beta	control network	-0.17772
Motor	Beta	compensatory	-0.12096
Motor	Beta	motor control	-0.12392
Motor	Beta	motor task	-0.10515
Motor	Beta	executive functions	-0.19263
Motor	Beta	motor response	-0.15881
Motor	Gamma	primary motor	-0.32019
Motor	Gamma	somatosensory	-0.30906
Motor	Gamma	speech perception	-0.282
Motor	Gamma	sensations	-0.2786
Motor	Gamma	motor control	-0.25469
Motor	Gamma	execution	-0.24586
Motor	Gamma	motor performance	-0.23014
Motor	Gamma	reorgnization	-0.23003
Motor	Gamma	multisensory	-0.22276
Motor	Gamma	sensorimotor network	-0.20673

3.6. Prediction analysis feature selection frequency by LASSO regression. The stability of feature selection in predictive models (classification and regression) was assessed using LASSO-regularized linear regression. Over 100 random shuffling repetitions,

features retaining non-zero coefficients were identified. The resulting figure displays the frequency/probability of each feature's selection across all repetitions.

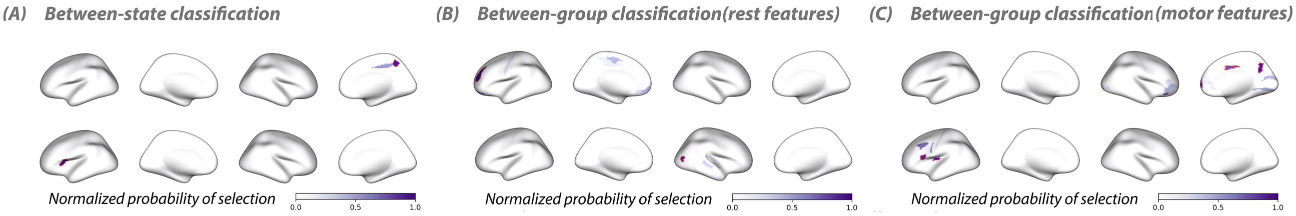

Fig. S7. Classification analysis feature selection

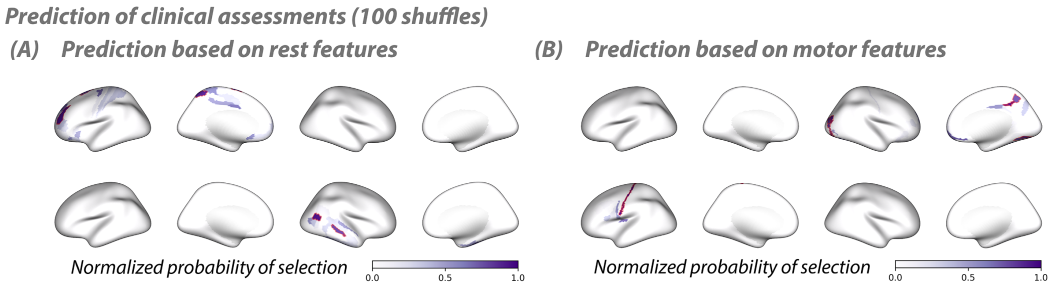

Fig. S8. Regression analysis feature selection

3.7. GO Statistics.

Table S4. Beta

ID	Description	GeneRatio	p.adjust	qvalue
GO:0023061	signal release	43/650	0.0001	0.0001
GO:0034765	regulation of monoatomic ion transmembrane transport	38/650	0.0011	0.001
GO:0046888	negative regulation of hormone secretion	12/650	0.0041	0.0038
GO:0071805	potassium ion transmembrane transport	22/650	0.0071	0.0066
GO:0050709	negative regulation of protein secretion	12/650	0.0071	0.0066
GO:0051224	negative regulation of protein transport	16/650	0.0071	0.0066
GO:0045055	regulated exocytosis	22/650	0.0071	0.0066
GO:0033555	multicellular organismal response to stress	13/650	0.0071	0.0066
GO:1904950	negative regulation of establishment of protein localization	16/650	0.0071	0.0066
GO:1903531	negative regulation of secretion by cell	17/650	0.0092	0.0085
GO:0019932	second-messenger-mediated signaling	26/650	0.0109	0.0101
GO:0006887	exocytosis	28/650	0.0109	0.0101
GO:0006816	calcium ion transport	33/650	0.0109	0.0101
GO:0006813	potassium ion transport	22/650	0.0109	0.0101
GO:0051260	protein homooligomerization	19/650	0.0133	0.0123
GO:0046676	negative regulation of insulin secretion	8/650	0.0155	0.0144
GO:0050796	regulation of insulin secretion	17/650	0.0155	0.0144
GO:0030073	insulin secretion	19/650	0.0157	0.0145
GO:0051259	protein complex oligomerization	22/650	0.0187	0.0173
GO:0070588	calcium ion transmembrane transport	27/650	0.021	0.0195
GO:0051048	negative regulation of secretion	17/650	0.021	0.0195
GO:0051216	cartilage development	19/650	0.021	0.0195
GO:0045176	apical protein localization	5/650	0.021	0.0195
GO:0050806	positive regulation of synaptic transmission	17/650	0.021	0.0195
GO:0009914	hormone transport	25/650	0.021	0.0195
GO:0090278	negative regulation of peptide hormone secretion	8/650	0.021	0.0195

Continued on next page

Table S4 – continued from previous page

ID	Description	GeneRatio	p.adjust	qvalue
GO:0007269	neurotransmitter secretion	15/650	0.021	0.0195
GO:0099643	signal release from synapse	15/650	0.021	0.0195
GO:0071241	cellular response to inorganic substance	20/650	0.021	0.0195
GO:0022604	regulation of cell morphogenesis	21/650	0.021	0.0195
GO:0002792	negative regulation of peptide secretion	8/650	0.0216	0.02
GO:0010721	negative regulation of cell development	23/650	0.0216	0.02
GO:0046883	regulation of hormone secretion	21/650	0.0216	0.02
GO:0002062	chondrocyte differentiation	13/650	0.0216	0.02
GO:0046879	hormone secretion	24/650	0.0216	0.02
GO:0048736	appendage development	17/650	0.0216	0.02
GO:0060173	limb development	17/650	0.0216	0.02
GO:0071277	cellular response to calcium ion	11/650	0.0216	0.02
GO:0050804	modulation of chemical synaptic transmission	33/650	0.0216	0.02
GO:0009306	protein secretion	27/650	0.0216	0.02
GO:0071248	cellular response to metal ion	18/650	0.0216	0.02
GO:0099177	regulation of trans-synaptic signaling	33/650	0.0216	0.02
GO:1903828	negative regulation of protein localization	19/650	0.0216	0.02
GO:0035592	establishment of protein localization to extracellular region	27/650	0.0216	0.02
GO:0009410	response to xenobiotic stimulus	30/650	0.0258	0.0239
GO:0030072	peptide hormone secretion	20/650	0.0267	0.0247
GO:0032386	regulation of intracellular transport	25/650	0.0267	0.0247
GO:0001505	regulation of neurotransmitter levels	17/650	0.0271	0.0251
GO:0071692	protein localization to extracellular region	27/650	0.0273	0.0253
GO:0015833	peptide transport	21/650	0.0284	0.0262
GO:0002790	peptide secretion	20/650	0.0319	0.0295
GO:0061448	connective tissue development	22/650	0.0328	0.0303
GO:0050708	regulation of protein secretion	21/650	0.0328	0.0303
GO:0051960	regulation of nervous system development	31/650	0.0331	0.0306
GO:0032330	regulation of chondrocyte differentiation	8/650	0.0356	0.0329
GO:0090276	regulation of peptide hormone secretion	17/650	0.0356	0.0329
GO:0050767	regulation of neurogenesis	27/650	0.0399	0.0369
GO:0002791	regulation of peptide secretion	17/650	0.041	0.0379
GO:1902075	cellular response to salt	17/650	0.0427	0.0395
GO:0090087	regulation of peptide transport	17/650	0.0444	0.0411
GO:0097501	stress response to metal ion	5/650	0.0464	0.0429
GO:0010959	regulation of metal ion transport	27/650	0.05	0.0462

Table S5. Gamma

ID	Description	GeneRatio	p.adjust	qvalue
GO:0030073	insulin secretion	25/648	0.0001	0.0001
GO:0002790	peptide secretion	27/648	0.0002	0.0002
GO:0050796	regulation of insulin secretion	21/648	0.0002	0.0002
GO:0030072	peptide hormone secretion	26/648	0.0002	0.0002
GO:0023061	signal release	40/648	0.0002	0.0002
GO:0002791	regulation of peptide secretion	23/648	0.0002	0.0002
GO:0015833	peptide transport	27/648	0.0002	0.0002
GO:0090087	regulation of peptide transport	23/648	0.0002	0.0002
GO:0046883	regulation of hormone secretion	26/648	0.0003	0.0003
GO:0009914	hormone transport	30/648	0.0003	0.0003
GO:0046879	hormone secretion	29/648	0.0004	0.0004
GO:0090276	regulation of peptide hormone secretion	22/648	0.0004	0.0004
GO:0009306	protein secretion	32/648	0.0005	0.0005
GO:0035592	establishment of protein localization to extracellular region	32/648	0.0005	0.0005
GO:0071805	potassium ion transmembrane transport	23/648	0.0005	0.0005

Continued on next page

Table S5 – continued from previous page

ID	Description	GeneRatio	p.adjust	qvalue
GO:0050708	regulation of protein secretion	26/648	0.0005	0.0005
GO:0051960	regulation of nervous system development	37/648	0.0006	0.0005
GO:0006882	intracellular zinc ion homeostasis	9/648	0.0006	0.0006
GO:0071692	protein localization to extracellular region	32/648	0.0006	0.0006
GO:0002792	negative regulation of peptide secretion	10/648	0.0006	0.0006
GO:0097501	stress response to metal ion	7/648	0.0009	0.0008
GO:0050767	regulation of neurogenesis	32/648	0.0011	0.001
GO:0042886	amide transport	30/648	0.0011	0.001
GO:0008277	regulation of G protein-coupled receptor signaling pathway	17/648	0.0011	0.001
GO:0046676	negative regulation of insulin secretion	9/648	0.0012	0.0011
GO:0010273	detoxification of copper ion	6/648	0.0016	0.0015
GO:1990169	stress response to copper ion	6/648	0.0016	0.0015
GO:0006813	potassium ion transport	23/648	0.0017	0.0015
GO:0022604	regulation of cell morphogenesis	23/648	0.0024	0.0022
GO:0046888	negative regulation of hormone secretion	11/648	0.0024	0.0022
GO:0090278	negative regulation of peptide hormone secretion	9/648	0.0027	0.0025
GO:0071248	cellular response to metal ion	20/648	0.0028	0.0025
GO:0010721	negative regulation of cell development	25/648	0.0029	0.0026
GO:0006816	calcium ion transport	33/648	0.0041	0.0037
GO:0050709	negative regulation of protein secretion	11/648	0.0055	0.005
GO:0010975	regulation of neuron projection development	33/648	0.0055	0.005
GO:0034765	regulation of monoatomic ion transmembrane transport	33/648	0.0056	0.0051
GO:1903531	negative regulation of secretion by cell	16/648	0.0084	0.0077
GO:0061687	detoxification of inorganic compound	6/648	0.0087	0.008
GO:0051048	negative regulation of secretion	17/648	0.0106	0.0097
GO:0140115	export across plasma membrane	11/648	0.0122	0.0112
GO:0045055	regulated exocytosis	20/648	0.0126	0.0115
GO:0071294	cellular response to zinc ion	6/648	0.0132	0.012
GO:1905145	cellular response to acetylcholine	7/648	0.0132	0.012
GO:0071241	cellular response to inorganic substance	20/648	0.0132	0.012
GO:0002275	myeloid cell activation involved in immune response	12/648	0.0132	0.012
GO:0051017	actin filament bundle assembly	16/648	0.0134	0.0123
GO:0051224	negative regulation of protein transport	14/648	0.0134	0.0123
GO:0009084	glutamine family amino acid biosynthetic process	5/648	0.014	0.0127
GO:0007015	actin filament organization	32/648	0.0143	0.013
GO:0032418	lysosome localization	11/648	0.0149	0.0136
GO:1990849	vacuolar localization	11/648	0.0149	0.0136
GO:1902075	cellular response to salt	18/648	0.0155	0.0141
GO:0061572	actin filament bundle organization	16/648	0.0155	0.0141
GO:0019932	second-messenger-mediated signaling	24/648	0.0155	0.0141
GO:0006887	exocytosis	26/648	0.0155	0.0141
GO:1904950	negative regulation of establishment of protein localization	14/648	0.0155	0.0141
GO:1903828	negative regulation of protein localization	19/648	0.0155	0.0141
GO:0071280	cellular response to copper ion	6/648	0.0158	0.0145
GO:0070588	calcium ion transmembrane transport	26/648	0.0168	0.0153
GO:0071276	cellular response to cadmium ion	7/648	0.0168	0.0153
GO:1905144	response to acetylcholine	7/648	0.0168	0.0153
GO:0021537	telencephalon development	22/648	0.0168	0.0153
GO:0009065	glutamine family amino acid catabolic process	6/648	0.0182	0.0166
GO:0007266	Rho protein signal transduction	14/648	0.0182	0.0166
GO:0098659	inorganic cation import across plasma membrane	14/648	0.0182	0.0166
GO:0099587	inorganic ion import across plasma membrane	14/648	0.0182	0.0166
GO:0033555	multicellular organismal response to stress	11/648	0.0189	0.0172
GO:0010038	response to metal ion	26/648	0.0199	0.0181
GO:0010043	response to zinc ion	8/648	0.0208	0.019
GO:0008360	regulation of cell shape	14/648	0.0212	0.0193

Continued on next page

Table S5 – continued from previous page

ID	Description	GeneRatio	p.adjust	qvalue
GO:0010977	negative regulation of neuron projection development	14/648	0.0212	0.0193
GO:0098754	detoxification	15/648	0.0228	0.0208
GO:0098739	import across plasma membrane	18/648	0.0238	0.0217
GO:0030038	contractile actin filament bundle assembly	12/648	0.0238	0.0217
GO:0043149	stress fiber assembly	12/648	0.0238	0.0217
GO:1902074	response to salt	26/648	0.024	0.0219
GO:0048260	positive regulation of receptor-mediated endocytosis	8/648	0.0245	0.0224
GO:0007264	small GTPase mediated signal transduction	32/648	0.0246	0.0225
GO:0051592	response to calcium ion	14/648	0.0254	0.0232
GO:0016358	dendrite development	19/648	0.0255	0.0233
GO:0051962	positive regulation of nervous system development	22/648	0.0255	0.0233
GO:0043303	mast cell degranulation	8/648	0.0262	0.024
GO:0002573	myeloid leukocyte differentiation	19/648	0.0276	0.0251
GO:0051259	protein complex oligomerization	20/648	0.0287	0.0262
GO:1902946	protein localization to early endosome	4/648	0.0287	0.0262
GO:1905668	positive regulation of protein localization to endosome	4/648	0.0287	0.0262
GO:0051056	regulation of small GTPase mediated signal transduction	22/648	0.03	0.0274
GO:0002279	mast cell activation involved in immune response	8/648	0.0315	0.0287
GO:0002695	negative regulation of leukocyte activation	17/648	0.0317	0.029
GO:0095500	acetylcholine receptor signaling pathway	6/648	0.0339	0.031
GO:0002448	mast cell mediated immunity	8/648	0.0344	0.0314
GO:0050866	negative regulation of cell activation	18/648	0.0353	0.0322
GO:0006883	intracellular sodium ion homeostasis	5/648	0.0365	0.0334
GO:0043650	dicarboxylic acid biosynthetic process	4/648	0.0365	0.0334
GO:1905666	regulation of protein localization to endosome	4/648	0.0365	0.0334
GO:0071277	cellular response to calcium ion	10/648	0.0371	0.0338
GO:0055078	sodium ion homeostasis	7/648	0.0424	0.0387
GO:0045807	positive regulation of endocytosis	14/648	0.0429	0.0391
GO:0031345	negative regulation of cell projection organization	16/648	0.0453	0.0414
GO:0098883	synapse pruning	4/648	0.0473	0.0432
GO:0051260	protein homooligomerization	16/648	0.0492	0.0449
GO:0030224	monocyte differentiation	6/648	0.0492	0.0449

3.8. KEGG Pathway Statistics.

Table S6. Beta

subcategory	ID	Description	GeneRatio	p.adjust	qvalue
Signal transduction	hsa04020	Calcium signaling pathway	22/309	0.0097	0.0089
Endocrine system	hsa04921	Oxytocin signaling pathway	15/309	0.0267	0.0246

Table S7. Gamma

subcategory	ID	Description	GeneRatio	p.adjust	qvalue
Digestive system	hsa04978	Mineral absorption	13/323	<0.0001	<0.0001
Development and regeneration	hsa04360	Axon guidance	20/323	0.0007	0.0006
Nervous system	hsa04724	Glutamatergic synapse	14/323	0.0034	0.0029
Nervous system	hsa04728	Dopaminergic synapse	15/323	0.0034	0.0029
Signal transduction	hsa04020	Calcium signaling pathway	22/323	0.0037	0.0031
Excretory system	hsa04964	Proximal tubule bicarbonate reclamation	6/323	0.0049	0.0041
Environmental adaptation	hsa04713	Circadian entrainment	12/323	0.005	0.0042
Nervous system	hsa04727	GABAergic synapse	11/323	0.0084	0.0071
Digestive system	hsa04971	Gastric acid secretion	10/323	0.0086	0.0072
Signal transduction	hsa04066	HIF-1 signaling pathway	12/323	0.0113	0.0095

Continued on next page

Table S7 – continued

subcategory	ID	Description	GeneRatio	p.adjust	qvalue
Excretory system	hsa04961	Endocrine and other factor-regulated calcium reabsorption	8/323	0.0113	0.0095
Nervous system	hsa04725	Cholinergic synapse	12/323	0.0156	0.0132
Endocrine system	hsa04911	Insulin secretion	10/323	0.0163	0.0137
Amino acid metabolism	hsa00220	Arginine biosynthesis	5/323	0.0203	0.0172
Signal transduction	hsa04371	Apelin signaling pathway	13/323	0.021	0.0177
Excretory system	hsa04960	Aldosterone-regulated sodium reabsorption	6/323	0.0325	0.0274
Nervous system	hsa04726	Serotonergic synapse	11/323	0.0335	0.0283
Nervous system	hsa04720	Long-term potentiation	8/323	0.0335	0.0283
Endocrine system	hsa04921	Oxytocin signaling pathway	13/323	0.0416	0.0351
Cell growth and death	hsa04216	Ferroptosis	6/323	0.044	0.0372

4. Supplementary Note

4.1. Interpretative scope of gene enrichment analysis. An important consideration regarding the gene enrichment analysis is its interpretative scope. The PLS regression and subsequent enrichment results should be viewed as a descriptive validation rather than providing mechanistic or causal evidence for the observed electrophysiological changes. Instead, this analysis serves as a tool to assess whether the spatial patterns of our neurophysiological findings align with known biological markers of PD. As this is the first study to apply functional gradient mapping to electrophysiological data in a PD cohort, this biological validation step is essential to ensure that the identified topographical features are indeed disease relevant. The PLS analysis utilizes a normative transcriptomic dataset to map the molecular vulnerability of specific cortical networks. This approach, widely established in the fMRI literature,49,50 identifies whether macroscale functional reorganization occurs in regions naturally enriched for PD-relevant molecular systems. By demonstrating that the topographical distribution of our results is spatially associated with the expression patterns of genes involved in PD-related pathophysiology, we provide evidence that the cortical gradient changes identified here align with the disease-related transcriptomic landscape. This alignment helps confirm that our findings are biologically grounded and specifically linked to PD-related pathology rather than reflecting non-specific or generalized neurodegeneration.

5. Study Limitation

This study has several limitations that should be considered. First, the small sample size of 35 PD patients and 34 healthy controls, while comparable to similar neuroimaging studies, may limit statistical power and generalizability. Second, although tremor-dominant phenotypes were represented in our cohort (approximately 26%), the current study primarily reflects the cortical organization of medication-responsive patients. We acknowledge that our study was conducted exclusively in the ON-medication state, limiting our ability to assess how dopamine replacement therapy directly modulates functional gradients. The decision to record in the ON state was prioritized to ensure high data quality by minimizing severe motor artifacts (e.g., tremor), which would otherwise confound high-density EEG source estimation. Recent fMRI evidence suggests that dopaminergic medication exerts a normalization effect on functional gradients[50 51], implying that the alterations we observed likely represent a conservative estimate of the underlying disease-related reorganization. Furthermore, given that adaptive DBS is clinically implemented in patients who remain on stable medication regimens, identifying robust biomarkers in the ON state is essential for translational applications. Future studies including an OFF-medication condition will be critical to further disentangle the specific effects of dopamine on cortical hierarchy.

In addition, the current study focused exclusively on motor symptoms and did not incorporate non-motor or cognitive clinical variables. Crucially, our cohort did not include any patients diagnosed with Parkinson's disease dementia or other clinically significant cognitive impairment. While exploring the alpha frequency range is theoretically valuable given its link to non-motor dysfunction, the lack of detailed cognitive clinical variables in this cohort limits the scope for identifying meaningful alpha-band correlates. To maintain the focus on established motor-frequency biomarkers (beta and gamma) for motor symptom management, we have provided exploratory alpha results in the Supplementary Material. The investigation of non-motor and cognitive clinical correlates remains an important objective for future study.

Regarding our experimental paradigm, a limitation is the state classification from an eyes-closed resting state to an eyes-open motor task. The choice of eyes-closed rest was intended to minimize eye-movement artifacts and capture intrinsic connectivity during the resting state; however, this may introduce a visual confound that likely contributed to the high accuracy of our state classification models. While our primary findings regarding disease-specific alterations and clinical predictions were derived from single-state analyses (thereby mitigating this confound), the lack of a dedicated resting eyes-open control group prevents a complete isolation of task-specific motor reorganization from the effects of visual arousal. Future investigations should incorporate an REO condition to further disentangle these distinct physiological components.

Finally, a methodological limitation concerns the alignment template for diffusion embedding in the gradient analysis. Although this study is the first to apply this technique to EEG data in PD, the gradient alignment template was derived

from the control group within the current study, rather than from a large, independent cohort of healthy subjects—such as the gradient templates from HCP datasets used in fMRI studies via the BrainSpace toolbox. This may limit the direct comparability of gradient patterns to normative data and introduce bias if the control group is not fully representative. To benefit the research community and facilitate future studies, the control group template generated in this work is available online and can be used as a reference for other groups conducting gradient analysis in similar contexts.

References

1. Vorderwülbecke Bernd J, Carboni Margherita, Tourbier Sebastien, et al. High-density Electric Source Imaging of interictal epileptic discharges: How many electrodes and which time point? *Clinical Neurophysiology.* 2020;131:2795–2803.
2. Delorme Arnaud, Makeig Scott. EEGLAB: an open source toolbox for analysis of single-trial EEG dynamics including independent component analysis *Journal of neuroscience methods.* 2004;134:9–21.
3. Makeig Scott, Bell Anthony J., Jung Tzyy-Ping, Sejnowski Terrence J.. Independent component analysis of electroencephalographic data in *Proceedings of the 9th International Conference on Neural Information Processing Systems*NIPS'95:145–151MIT Press 1995.
4. Lee Te-Won, Girolami Marc, Sejnowski Terrence J.. Independent component analysis using an extended infomax algorithm for mixed subgaussian and supergaussian sources *Neural Computation.* 1999;11:417–441.
5. Viola Francesco C., Thorne John, Edmonds Ben, Schneider Thomas, Eichele Tom, Debener Stefan. Semi-automatic identification of independent components representing EEG artifact *Clinical Neurophysiology.* 2009;120:868–877.
6. Pion-Tonachini Luca, Kreutz-Delgado Ken, Makeig Scott. ICLabel: An automated electroencephalographic independent component classifier, dataset, and website *NeuroImage.* 2019;198:181–197.
7. Friston Karl. Statistical Parametric Mapping. Part 7: Connectivity in *Statistical Parametric Mapping*:541–560 2007.
8. Gaser Christian, Dahnke Robert, Thompson Paul M., Kurth Florian, Luders Eileen, Alzheimer's Disease Neuroimaging Initiative . CAT: a computational anatomy toolbox for the analysis of structural MRI data *GigaScience.* 2024;13:giae049.
9. Tadel Francois, Baillet Sylvain, Mosher John C., Pantazis Dimitrios, Leahy Richard M.. Brainstorm: A User-Friendly Application for MEG/EEG Analysis *Computational Intelligence and Neuroscience.* 2011;2011:879716.
10. Nielsen Jonas D., Madsen Kasper H., Puonti Olli, et al. Automatic skull segmentation from MR images for realistic volume conductor models of the head: Assessment of the state-of-the-art *NeuroImage.* 2018;174:587–598.
11. Thielscher Axel, Antunes Andre, Saturnino Gabrio B.. Field modeling for transcranial magnetic stimulation: A useful tool to understand the physiological effects of TMS? in *2015 37th Annual International Conference of the IEEE Engineering in Medicine and Biology Society (EMBC)*:222–225 2015.
12. Schrader Stefan, Westhoff Annika, Piastra Maria C., et al. DUNEuro—A software toolbox for forward modeling in bioelectromagnetism *PLOS ONE.* 2021;16:e0252431.
13. Ding Hong, Medani Taha, Tadel Francois. Brainstorm Forum: Headmodel with EEG questions 2021. Accessed: February 26, 2025.
14. Van Veen Barry D., van Drongelen Wim, Yuchtman Marc, Suzuki Akihiro. Localization of brain electrical activity via linearly constrained minimum variance spatial filtering *IEEE Transactions on Biomedical Engineering.* 1997;44:867–880.
15. Maćkiewicz Andrzej, Ratajczak Wacław. Principal components analysis (PCA) *Computers and Geosciences.* 1993;19:303–342.
16. Glasser Matthew F, Coalson Timothy S, Robinson Emma C, et al. A multi-modal parcellation of human cerebral cortex *Nature.* 2016;536:171–178.
17. Šverko Zvonimir, Vrankić Matea, Vlahinić Saša, Rogelj Peter. Complex Pearson Correlation Coefficient for EEG Connectivity Analysis *Sensors.* 2022;22:1477.
18. Van der Wael Ruben, Benkarim Oualid, Paquola Carolina, et al. BrainSpace: a toolbox for the analysis of macroscale gradients in neuroimaging and connectomics datasets *Communications Biology.* 2020;3:103.
19. Margulies Daniel S., Ghosh Satrajit S., Goulas Alexandros, et al. Situating the default-mode network along a principal gradient of macroscale cortical organization *Proceedings of the National Academy of Sciences.* 2016;113:12574–12579.
20. Xie Kui, Royer Julien, Larivière Stéphanie, et al. Atypical connectome topography and signal flow in temporal lobe epilepsy *Progress in Neurobiology.* 2024;236:102604.
21. Park Bomi, Van der Wael Ruben, Paquola Carolina, et al. Signal diffusion along connectome gradients and inter-hub routing differentially contribute to dynamic human brain function *NeuroImage.* 2021;224:117429.
22. Ah-Pine Jean. Advances in Knowledge Discovery and Data Mining in *Lecture Notes in Computer Science*:362–373 2010.
23. Coifman Ronald R., Lafon Stephane, Lee Ann B., et al. Geometric diffusions as a tool for harmonic analysis and structure definition of data: Diffusion maps *Proceedings of the National Academy of Sciences.* 2005;102:7426–7431.
24. Larivière Stéphanie, Bayrak Şebnem, Van der Wael Ruben, et al. BrainStat: A toolbox for brain-wide statistics and multimodal feature associations *NeuroImage.* 2023;266:119807.
25. Ji John L., Spronk Matthijs, Kulkarni Kaustubh, Repovš Grega, Anticevic Alan, Cole Michael W.. Mapping the human brain's cortical-subcortical functional network organization *NeuroImage.* 2019;185:35–57.
26. Benjamini Yoav, Hochberg Yosef. Controlling the False Discovery Rate: A Practical and Powerful Approach to Multiple Testing *Journal of the Royal Statistical Society: Series B (Methodological).* 1995;57:289–300.
27. De la Vega W. T.. NeuroSynth: a new platform for large-scale automated synthesis of human functional neuroimaging data *Frontiers in Neuroinformatics.* 2011;5.

28. Yarkoni Tal, Poldrack Russell A., Nichols Thomas E., Van Essen David C., Wager Tor D.. Large-scale automated synthesis of human functional neuroimaging data *Nature Methods.* 2011;8:665–670.
29. Zuo Xi-Nian, Ehmke Rick, Mennes Maarten, et al. Network Centrality in the Human Functional Connectome *Cerebral Cortex.* 2012;22:1862–1875.
30. Okuno Takahiro, Woodward Aaron. Vector Auto-Regressive Deep Neural Network: A Data-Driven Deep Learning-Based Directed Functional Connectivity Estimation Toolbox *Frontiers in Neuroscience.* 2021;15:764796.
31. Neath Andrew A., Cavanaugh Joseph E.. The Bayesian information criterion: background, derivation, and applications *Wiley Interdisciplinary Reviews: Computational Statistics.* 2012;4:199–203.
32. Kingma Diederik P., Ba Jimmy. Adam: A Method for Stochastic Optimization 2014.
33. Granger Clive W. J.. Investigating Causal Relations by Econometric Models and Cross-spectral Methods *Econometrica.* 1969;37:424–438.
34. Portnoy Stephen, Huber Peter J.. Robust Statistics *Technometrics.* 1982;24:163–176.
35. Griffis Joshua C., Bruss Joel, Acker Stephanie F., Shea Christine, Tranel Daniel, Boes Aaron D.. Iowa Brain-Behavior Modeling Toolkit: An Open-Source MATLAB Tool for Inferential and Predictive Modeling of Imaging-Behavior and Lesion-Deficit Relationships *Human Brain Mapping.* 2024;45:e70115.
36. Krstajic Daniel, Buturovic Ljubomir J., Leahy David E., Thomas Shane. Cross-validation pitfalls when selecting and assessing regression and classification models *Journal of Cheminformatics.* 2014;6:10.
37. Parvandeh Samira, Yeh H.W., Paulus Martin P., McKinney Brett A.. Consensus features nested cross-validation *Bioinformatics.* 2020;36:3093–3098.
38. Tenenbaum Joshua B., De Silva Vin, Langford John C.. A Global Geometric Framework for Nonlinear Dimensionality Reduction *Science.* 2000;290:2319–2323.
39. Tibshirani Robert. Regression Shrinkage and Selection Via the Lasso *Journal of the Royal Statistical Society: Series B (Statistical Methodology).* 1996;58:267–288.
40. Lee Cheol-Ho, Park Hye-Young, Lee Min-Jung, Park Bo-Young. Whole-brain functional gradients reveal cortical and subcortical alterations in patients with episodic migraine *Human Brain Mapping.* 2023;44:2224–2233.
41. Jones Allan R., Overly Clifford C., Sunkin Susannah M.. The Allen Brain Atlas: 5 years and beyond *Nature Reviews Neuroscience.* 2009;10:821–828.
42. Xu Ting, Chen Zhen, Zhou Xiaoyi, et al. The central renin–angiotensin system: A genetic pathway, functional decoding, and selective target engagement characterization in humans *Proceedings of the National Academy of Sciences.* 2024;121:e2306936121.
43. Li Jia, Seidlitz Jacob, Suckling John, et al. Cortical structural differences in major depressive disorder correlate with cell type-specific transcriptional signatures *Nature Communications.* 2021;12:1647.
44. Seidlitz Jacob, Nadig Anish, Liu Shenyang, et al. Transcriptomic and cellular decoding of regional brain vulnerability to neurogenetic disorders *Nature Communications.* 2020;11:3358.
45. Xiao Yulong, Zhao Lihong, Zang Xiaoying, Xue Shuan. Compressed primary-to-transmodal gradient is accompanied with subcortical alterations and linked to neurotransmitters and cellular signatures in major depressive disorder *Human Brain Mapping.* 2023;44:5919–5935.
46. Whitaker Kirstie J., Vértes Petra E., Romero-Garcia Rafael, et al. Adolescence is associated with genomically patterned consolidation of the hubs of the human brain connectome *Proceedings of the National Academy of Sciences.* 2016;113:9105–9110.
47. de Bézenac Camille E., Caciagli Lorenzo, Alonazi Badr K., Bernhardt Boris C., Marson Anthony G., Keller Simon S.. Altered functional connectome hierarchy with gene expression signatures in newly-diagnosed focal epilepsy 2021.
48. Abdi Hervé, Williams Lynne J.. Partial least squares methods: partial least squares correlation and partial least square regression in *Methods in Molecular Biology*;930:549–579 2012.
49. Yu Guangchuang, Wang Li-Gen, Han Yanyan, He Qing-Yu. clusterProfiler: an R Package for Comparing Biological Themes Among Gene Clusters *OMICS: A Journal of Integrative Biology.* 2012;16:284–287.
50. Guo Tao, Xuan Min, Zhou Cheng, et al. Normalization effect of levodopa on hierarchical brain function in Parkinson's disease *Network Neuroscience.* 2022;6:552–569.
51. Wu Chenqing, Wu Haoting, Zhou Cheng, et al. Normalization effect of dopamine replacement therapy on brain functional connectome in Parkinson's disease *Human Brain Mapping.* 2023;44:3845–3858.
